# Supplementary material for: A guide to using a multiple-matrix animal model to disentangle genetic and nongenetic causes of phenotypic variance
Source: PLoS One. 2018 Oct 12;13(10):e0197720. doi: 10.1371/journal.pone.0197720 (PMC6193571; doi:10.1371/journal.pone.0197720)
Supplement: S1 File — Detailed tutorial for carrying out the analyses shown in this paper. (PDF) [file pone.0197720.s001.pdf]

# Asreml-r Tutorial

## Contents

**Simulated Data** 2

**Reading in the data** 2

**Setting up** 3

**Basic model** 6
Random effect significance . . . . . 7
Heritability . . . . . 7

**Including environmental similarity** 8
Body size and swimming speed . . . . . 10

**Parental effects** 11
Maternal environmental variance . . . . . 13
Maternal genetic variance . . . . . 14

**Including epiallelic similarity** 15

**Including a social network** 17
Including a weighted social network . . . . . 20

**Plotting the models** 21

**Using a genomic relatedness matrix** 25

**Running models in MCMCglmm** 25
Additive genetic relatedness . . . . . 26
Basic model . . . . . 26
Including environmental similarity . . . . . 29
Including parental environmental variance . . . . . 31

**References** 32

In this tutorial, we demonstrate how to run animal models that include non-genetic causes of similarity between individuals. This is shown using the proprietary R package ASREML-R, and so users must have a licence to use this software. For those who do not have a licence, we also have a brief tutorial for the same analyses in MCMCglmm at the end of this document.

For each of the models in the main article, we show how the data is prepared for analysis, and the code to run the models. Additionally, we then show how the proportion of variance explained by the different variance parameters can be calculated, including narrow sense heritability.

# Simulated Data

We have simulated a population of Mermaids, over 10 generations, with the population size varying at random between 150 and 250 individuals. Individuals reside in a lagoon defined by a 50x50 grid, over which 5 environmental measures vary. Here, and in the main text, we demonstrate the models using three traits that have all been simulated to have additive genetic and direct environmental effects. The second and third traits also include maternal enviromental effects, and the third trait also includes maternal genetic effects (for those who are interested in seeing how these were simulated in detail, we provide separate Data Simulation Documentation).

In order to show how other non-genetic factors could be used to account for variance in a trait, we have also simulated epigenetic differences between individuals, as well as a social network. In the first case, individuals vary in the number of methylated CpG loci at 230 CpG islands. Of these 230, 100 vary at random, 100 are based upon the environment, and 30 are based upon the environment, but are transmitted (or reconstructed) from mothers to offspring. Individuals inherit epigenetic states depending on the mother's environment, with a 50% probability of reset to a state determined by the individual's own environment

For the social network, we simulate interactions between individuals in the same generations, and between individuals in adjacent generations (e.g. those in generation 3 interact with individuals in generation 2, 3 and 4, but no others). Interactions are most likely within generations, particularly between those in close spatial proximity. Between generations, interactions are most likely between mother-offspring pairs, and again with weighting towards those closer together in space. Least likely are interactions between non-related pairs in separate environments (with this likelihood declining with distance).

Both the epigenetic information and the social network are simulated to follow the environment (and thus explain some variance in the trait if included in the model). However, the epigenetic and social information are not used to simulate traits directly.

# Reading in the data

The following code shows the data frames needed for this tutorial and loads the data that are needed. Modify the filepaths to where you have saved the relevant files. *Mermaids.csv* contains the pedigree, environmental, and phenotypic data. Columns *id*, *dam* and *sire* identify an individual, its mother, and its father. The column *generation* is an individual's generation/cohort, *sex* is 1=female or 0=male. Spatial coordinates are given by *Xloc* and *Yloc*, with *disp_dist* the distance an individual dispersed away from its mother. The three phenotypic traits are given by *trait_ae* (tail-fin colour), *trait_mee* (body size) and *trait_mgee* (swimming speed). Finally, the 5 environmental variables are in the columns *e_var_1* to *e_var_5*.

```
# libraries needed for these analyses
library(asreml)
library(nadiv)
library(igraph)
library(tnet)
library(pedantics)
```

```r
library(ggplot2)
library(MCMCglmm)
library(scales)


# File paths should be changed depending on where data is stored

# read in simulation data - pedigree and phenotypes
# telling R that there is a header row containing column names,
# and "NA" is the symbol for missing values
Mermaids<-read.csv("Mermaids.csv", header=TRUE, na.strings = "NA")

# read in epiallelic data
epigenetic<-read.csv("epigenetic.csv", row.names=1, header=TRUE, na.strings="NA")

# read in social network data
social<-as.matrix(read.csv("SocialNetwork.csv", row.names=1, na.strings="NA"))
```

The Mermaids data frame contains individual phenotype data, pedigree data, and environmental data:

```r
head(Mermaids)
```

```
##    id  dam sire generation sex Xloc Yloc disp_dist   trait_ae   trait_mee
## 1 ID1 <NA> <NA>          1   1    6   35        NA -0.7001089 -0.53448853
## 2 ID2 <NA> <NA>          1   0   38   11        NA -2.7462280 -3.16168191
## 3 ID3 <NA> <NA>          1   1   30   26        NA  0.4518192  1.35607602
## 4 ID4 <NA> <NA>          1   0    5    6        NA -1.7627434 -2.78136069
## 5 ID5 <NA> <NA>          1   1   24   18        NA -2.4402914 -2.81079338
## 6 ID6 <NA> <NA>          1   1   14   34        NA -0.5705943  0.06981653
##   trait_mgee    e_var_1    e_var_2     e_var_3    e_var_4     e_var_5
## 1 -0.2648912 -0.5122480  1.1630054  0.53553590 -0.5011063  0.77106100
## 2 -1.7269262 -0.9581736 -0.8660340 -0.56717393  1.0109815 -1.19462484
## 3  0.7195265  0.1109596 -3.1997209  0.07664997  1.2882459 -0.01460305
## 4 -4.1074544 -0.1372246  0.4657080 -1.43283140  0.8872330  0.54445023
## 5 -4.0411930  0.9605647 -1.3748215 -3.25305700 -0.6360135 -0.04752684
## 6 -0.3315978  1.6439259 -0.6472217 -0.41422627 -0.7636775 -0.20009279
```

## Setting up

To connect phenotypic and pedigree data, the pedigree columns must be factors (rather than characters). We also generate duplicates of these columns, so that they can be linked to the similarity matrices separately (from the environment, epialleles, or social network).

```r
# makes ID/dam factors
Mermaids$id<-as.factor(Mermaids$id)
Mermaids$dam<-as.factor(Mermaids$dam)
Mermaids$sire<-as.factor(Mermaids$sire)

# Need separate columns later to estimate additive and maternal genetic effects
Mermaids$ANIMAL<-as.factor(Mermaids$id)
Mermaids$MOTHER<-as.factor(Mermaids$dam)

# Repeat for connection to the extra matrices in the models
```

```r
Mermaids$ANIMAL2<-as.factor(Mermaids$id)
Mermaids$MOTHER2<-as.factor(Mermaids$dam)

# For individuals in the first generation in the data
# no information is available on their parentage

# Need to ensure other values are correctly read in as numeric/factors
Mermaids$trait_ae<-as.numeric(Mermaids$trait_ae)
Mermaids$trait_mee<-as.numeric(Mermaids$trait_mee)
Mermaids$trait_mgee<-as.numeric(Mermaids$trait_mgee)

Mermaids$sex<-as.factor(Mermaids$sex)
Mermaids$gener<-as.factor(Mermaids$gener)

# Can be done for all the factors in the data,
# but not needed for this particular analysis
```

In order to run an animal model we need to provide relatedness information to the model. This is derived from the pedigree data in this case (genomic relatedness matrices are also increasingly being used, see page 25). Asreml-r requires the inverse of the relatedness matrix between individuals:

```r
# gets pedigree inverse for the model

# first get the pedigree from the data
# asreml-r expects this in the order "id","sire,"dam""
pedigree<-Mermaids[,c("id","sire","dam")]


# inserts any missing individuals into the pedigree

missing<-unique(Mermaids$id[which(!Mermaids$id%in%pedigree[,1])])
if(length(missing)>0){
  pedigree<-insertPed(pedigree, missing)
}

# Orders the pedigree so that parents come before their offspring
pedigree<-orderPed(pedigree)

# asreml.Ainverse then creates the inverse of the additive genetic relationship matrix
# from the pedigree data
ainv<-asreml.Ainverse(pedigree)$ginv
```

We can visualise the (raw) pedigree using the drawPedigree function from the Pedantics package:

```r
# drawPedigree expects "id" "dam" "sire"
# so change order of columns
drawPedigree(pedigree[,c(1,3,2)])
```

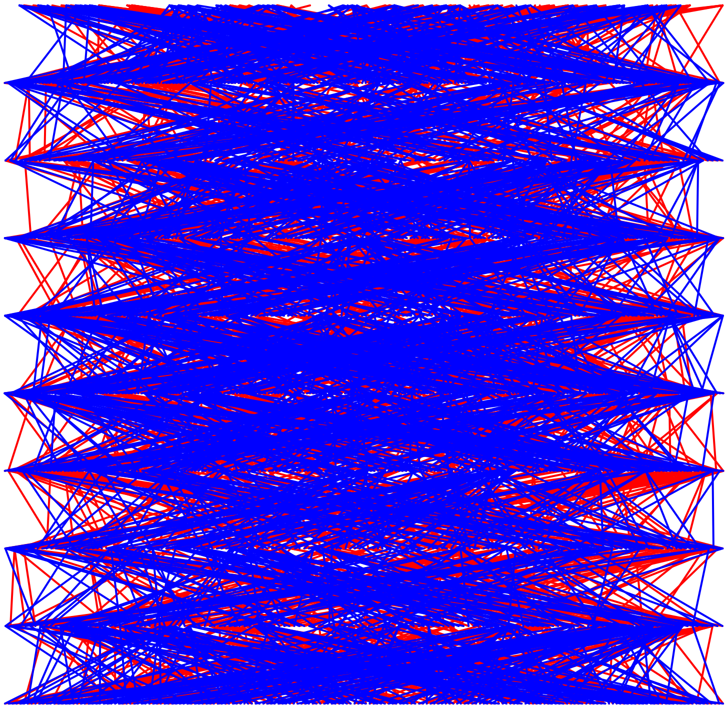

Alternatively, we can visualise the pedigree in 3D space, so that dispersal can be seen. Here, the physical locations of individuals are plotted on the X and Y axes (locations on spatial grid). The Z-axis shows the generations (with the 1st generation at the bottom of the axis, and the 10th generation at the top). Individual points are coloured by sex (blue for males, red for females). Lines connect offspring to their mothers (red lines) and fathers (blue lines). Note that the image we show below is a 2D capture of the full 3D plot - running the code below will produce a moveable plot wherein details are more evident.

```
library(rgl)

colors <- Mermaids$sex
colors[which(colors==1)]<- "red"
colors[which(colors==0)]<- "blue"


plot3d(Mermaids$Xloc,Mermaids$Yloc,Mermaids$generation,
       type="p", size=5, col=colors,
       xlab="X", ylab="Y", zlab="Generation")


for(i in min(which(Mermaids$generation>1)):nrow(Mermaids)){
  dam<-Mermaids$dam[i]
  sire<-Mermaids$sire[i]
  x1<-Mermaids$Xloc[i]
  x2<-Mermaids$Xloc[which(as.character(Mermaids$id)==dam)]
  x3<-Mermaids$Xloc[which(as.character(Mermaids$id)==sire)]
  y1<-Mermaids$Yloc[i]
  y2<-Mermaids$Yloc[which(as.character(Mermaids$id)==dam)]
  y3<-Mermaids$Yloc[which(as.character(Mermaids$id)==sire)]
  z1<-Mermaids$generation[i]
  z2<-Mermaids$generation[which(as.character(Mermaids$id)==dam)]
```

```
  z3<-Mermaids$generation[which(as.character(Mermaids$id)==sire)]

  lines3d(z= c(z1,z2), y = c(y1,y2), x = c(x1,x2), col = "firebrick", lwd =1)

  lines3d(z= c(z1,z3), y = c(y1,y3), x = c(x1,x3), col = "lightskyblue", lwd =1)
}
```

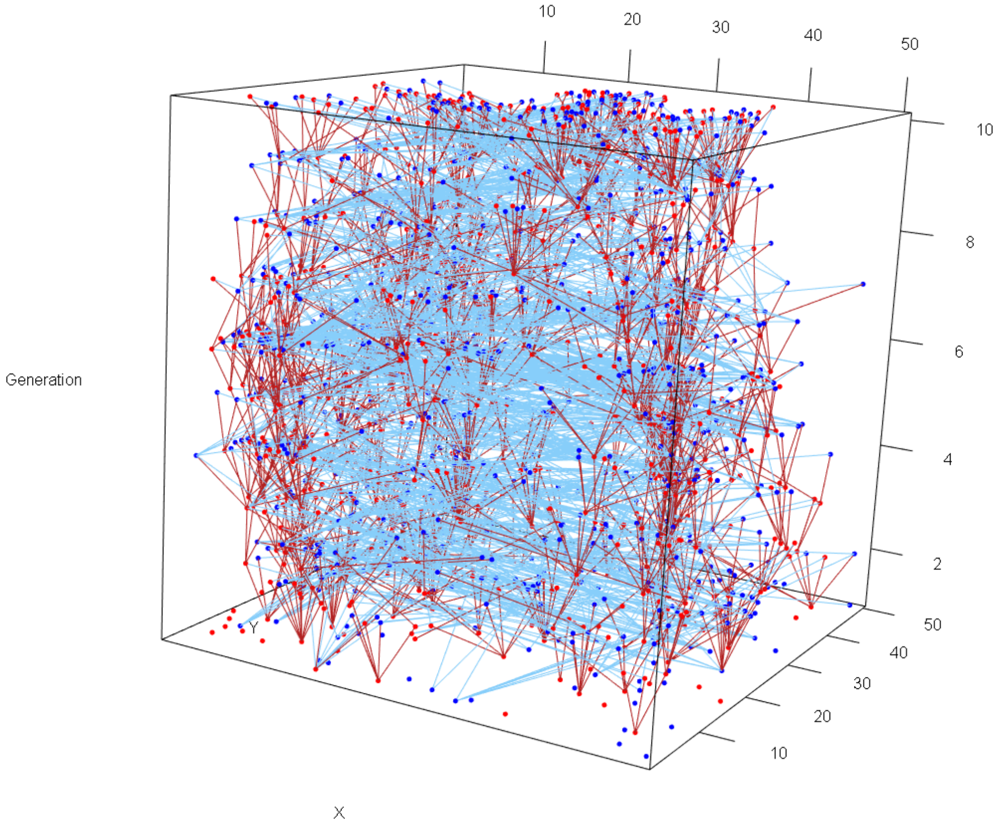


## Basic model

We show first a basic animal model, which includes only the additive genetic variance and residual variance, using the first phenotypic trait of tail-fin colour. Here, the only fixed effect is the mean/intercept of the reponse variable (*trait_ae ~ 1*). A single random effect (additive genetic) is included (*random= ~ ped(ANIMAL)*). The identity of the individual in the data is linked to the inverse relatedness matrix using the ginverse argument (*ginverse=list(ANIMAL=ainv)*). This model estimates additive genetic effect variance, assuming

that it has a mean of 0 and variance-covariance $\mathbf{A} * V_a$, such that the additive genetic effects are estimated according to the distribution $\mathbf{a} \sim N(0, \mathbf{A} * V_a)$. The covariance between individuals is determined by the additive genetic relatedness matrix ($\mathbf{A}$, derived from the pedigree),

```
# Basic model - estimates only additive genetic variance and residual variance

m_basic_1<-asreml(fixed=trait_ae ~ 1 ,
                  random= ~   ped(ANIMAL) ,
                  data=Mermaids,
                  ginverse=list(ANIMAL=ainv),
                  na.method.X="omit", na.method.Y="omit")
```

We can see the estimated variance components by calling

```
summary(m_basic_1)$varcomp
```

```
##                    gamma component std.error   z.ratio constraint
## ped(ANIMAL)!ped 1.164004  1.713575 0.1876646  9.131051   Positive
## R!variance      1.000000  1.472138 0.1096644 13.424029   Positive
```

which shows that there is a considerable amount of additive genetic variance in this trait (*ped(ANIMAL)!ped* = $V_a$=1.71), as well as residual variance (*R!variance* = $V_r$=1.47). In this model, we fitted just one random effect, and so the variance is partitioned into two components. The ratio of the value of a component to its standard error (the z-ratio) gives us an indication of the significance of the random effects. As the z-ratio of $V_a$ is greater than 2 (ie. the parameter is more that two standard errors from zero), it looks to be highly significant.

## Random effect significance

We can formally test the significance of random effects by rerunning the model with the random effect of interest removed, and comparing the log-likelihoods of the full and reduced model. We demonstrate this here for the above model only, but the same has been done to generate the tables of results for all the models we show in this tutorial (and has been done to generate the significance of random effects in the main paper).

```
m_basic_null<-asreml(fixed=trait_ae ~ 1 ,
                  data=Mermaids,
                  na.method.X="omit", na.method.Y="omit")
```

```
1-pchisq(2*(m_basic_1$loglik-m_basic_null$loglik),1)
```

```
## [1] 0
```

## Heritability

Heritability in the simplest case is calculated as the proportion of total variance explained by the additive genetic variance. As there are only two components of variance in this model, this is simply calculated as $V_a/(V_a + V_r)$. This can be done directly with the *nadiv* package in R to obtain both the estimate and the standard error for heritability:

```
nadiv:::pin(m_basic_1, herit ~ V1 / ( V1 + V2  ) )
```

```
##        Estimate         SE
## herit 0.5378937 0.04249887
```

Table 1 shows the absolute and proportional values of the variance explained for the additive genetic and residual variance, generated using the codes shown above.

Table S1: Variance estimates, and their proportions, for tail-fin colour from an animal model that contains only additive genetic ($V_a$) and residual ($V_r$) variance. Statistical significance of random effects is calculated using log-likelihood tests

| Component | Estimate | Standard Error | z-ratio | pvalue | Proportion | Proportion SE |
|---|---|---|---|---|---|---|
| Va | 1.714 | 0.1877 | 9.131 | <0.001 | 0.5379 | 0.0425 |
| Vr | 1.472 | 0.1097 | 13.424 | | 0.4621 | 0.0425 |

Doing the same models with **body size** gives:

```
# Basic model - includes only additive genetic variance

m_basic_2<-asreml(fixed=trait_mee ~ 1 ,
                  random= ~   ped(ANIMAL) ,
                  data=Mermaids,
                  ginverse=list(ANIMAL=ainv),
                  na.method.X="omit", na.method.Y="omit")
```

Table S2: Variance estimates, and their proportions, for body size from an animal model that contains only additive genetic ($V_a$) and residual ($V_r$) variance. Statistical significance of random effects is calculated using log-likelihood tests

| Component | Estimate | Standard Error | z-ratio | pvalue | Proportion | Proportion SE |
|---|---|---|---|---|---|---|
| Va | 1.730 | 0.1910 | 9.054 | <0.001 | 0.4855 | 0.04038 |
| Vr | 1.833 | 0.1199 | 15.280 | | 0.5145 | 0.04038 |

and for **swimming speed**:

```
m_basic_3<-asreml(fixed=trait_mgee ~ 1 ,
                  random= ~   ped(ANIMAL) ,
                  data=Mermaids,
                  ginverse=list(ANIMAL=ainv),
                  na.method.X="omit", na.method.Y="omit")
```

Table S3: Variance estimates, and their proportions, for swimming speed from an animal model that contains only additive genetic ($V_a$) and residual ($V_r$) variance. Statistical significance of random effects is calculated using log-likelihood tests

| Component | Estimate | Standard Error | z-ratio | pvalue | Proportion | Proportion SE |
|---|---|---|---|---|---|---|
| Va | 2.067 | 0.2151 | 9.611 | <0.001 | 0.4982 | 0.03841 |
| Vr | 2.082 | 0.1340 | 15.541 | | 0.5018 | 0.03841 |

# Including environmental similarity

In order to account for non-genetic similarity between individuals, we can fit additional random effects that account for variance in tail-fin colour. In the first instance, we show how this can be done using continuous environmental measures.

Each individual in the *Mermaids* data frame has 5 measurements corresponding to the 5 environmental variables, in the columns named *e_var_1* to *e_var_5*.

```
head(Mermaids[,c("id", "e_var_1", "e_var_2", "e_var_3", "e_var_4", "e_var_5")])
```

As environmental measures may occur on different scales, we first scale and center the environmental data. This is done by taking away the mean (so the mean becomes 0) and dividing by the standard deviation for each environmental measure (so the variance becomes 1).

```
# First gets the relevant columns from the data
enm<-Mermaids[,c("e_var_1", "e_var_2", "e_var_3", "e_var_4", "e_var_5")]

# Centers the data, and scales by standard deviation
enm<-scale(enm, center=TRUE, scale=TRUE)

rownames(enm)<-Mermaids$id
```

To obtain the environmental similarity between individuals, we first calculate the Euclidean distance between each pair of individuals in the data, calculated as $d_{(i,j)} = \sqrt{\sum_1^k (p_k - q_k)^2}$ where $p$ and $q$ are the scaled environmental measures (from environment measures 1 to $k$) of individuals $i$ and $j$. Note that this is not the physical distance between the locations of individuals in space. We then scale this matrix of distances so that they are between 0 and 1. There must be 1's on the diagonal, as an individual has an identical environment to itself. Values closer to 0 show individuals with more dissimilar environments. Thus elements of the matrix $\mathbf{S_n}$ are:

$$S_{n(i,j)} = 1 - \frac{d_{(i,j)}}{max(d)}$$

where $max(d)$ is the maximum euclidean distance found between all pairs of individuals.

```
# similarity matrix between individuals
# calculates the euclidean distance between each individual's environment parameters
p<-as.matrix(dist(enm, method="euclidean", diag=TRUE,upper=TRUE))

# then scales so that the values are between 0 and 1
p<-1-p/max(p, na.rm=TRUE)

# distances of NA need to be 0 if there are any
# no missing values in this data, so no NAs in the matrix
# p[which(is.na(p))]<-0
```

```
# showing here the first individuals
# note that values are 1 on the diagonal
# (rounded values displayed to make it easier to see)
round(p[1:10,1:10], digits=3)
```

```
##        ID1   ID2   ID3   ID4   ID5   ID6   ID7   ID8   ID9  ID10
## ID1  1.000 0.564 0.360 0.688 0.398 0.592 0.660 0.745 0.757 0.677
## ID2  0.564 1.000 0.619 0.683 0.517 0.570 0.847 0.727 0.454 0.550
## ID3  0.360 0.619 1.000 0.472 0.465 0.521 0.589 0.531 0.293 0.370
## ID4  0.688 0.683 0.472 1.000 0.587 0.620 0.793 0.865 0.574 0.778
## ID5  0.398 0.517 0.465 0.587 1.000 0.639 0.525 0.519 0.266 0.608
## ID6  0.592 0.570 0.521 0.620 0.639 1.000 0.578 0.650 0.539 0.719
## ID7  0.660 0.847 0.589 0.793 0.525 0.578 1.000 0.834 0.527 0.616
## ID8  0.745 0.727 0.531 0.865 0.519 0.650 0.834 1.000 0.663 0.732
## ID9  0.757 0.454 0.293 0.574 0.266 0.539 0.527 0.663 1.000 0.608
## ID10 0.677 0.550 0.370 0.778 0.608 0.719 0.616 0.732 0.608 1.000
```

We then invert the matrix using the *solve()* function so that the matrix inverse can be used in ASReml-R.

```
# gets the matrix inverse
p<-as(solve(p),"dgCMatrix")
p<-as.matrix(p)
```

We can include this matrix in the animal model in an analagous manner to including the additive genetic relatedness. The identities of individuals in the phenotypic data are linked to the inverse of the environmental similarity matrix by inlcuding it in the *ginverse*, and the random effect is specified as *giv(ANIMAL2)*.

Note that as these models take longer to converge, and require more memory to run, we have increased the *maxiter* (number of iterations), *workspace* and *pworkspace* arguments. As a consequence, these models may struggle to run on computers with limited memory. For example, the model containing only additive genetic variance takes around 3 seconds on a Windows 10 desktop with 32GB RAM and 3.40GHz CPU, using only around 2% of the computers CPU. The model below takes around 7 minutes, and uses uses around 15% of the CPU. As more random effects are included in the sections that follow, computing requirements increase futher.

For tail-fin colour, the model specification is:

```
m_env_1<-asreml(fixed=trait_ae ~ 1 ,
                 random= ~ ped(ANIMAL) + giv(ANIMAL2),
                 data=Mermaids,
                 ginverse=list(ANIMAL=ainv,
                               ANIMAL2=p),
                 na.method.X="omit", na.method.Y="omit",
                 workspace=300e6,pworkspace=300e6,
                 maxiter=50)
```

We can then see the variance components that have been estimated from this model (Table 3).

```
summary(m_env_1)$varcomp
```

```
##                        gamma component  std.error   z.ratio constraint
## ped(ANIMAL)!ped     1.230779 1.1003540 0.12400652  8.873356   Positive
## giv(ANIMAL2).giv    1.102948 0.9860696 0.21690876  4.546011   Positive
## R!variance          1.000000 0.8940308 0.07390975 12.096249   Positive
```

This model estimates each variance to be close to 1, which was the variance that was simulated. Thus, the variances in tail-fin colour are accurately estimated using this model. As is shown in the main manuscript (Figure 3), the inclusion of the environmental similarity matrix reduces the proportion of variance in additive genetic and residual components.

As before, we can estimate the proportion of variance explained by the different components. For heritability:

```
nadiv:::pin(m_env_1, herit ~ V1 / ( V1 + V2 + V3 ) )
```

```
##        Estimate         SE
## herit   0.36919 0.04191195
```

Giving a heritability close to the expected value of 0.33 (i.e. 1/3 of the total variance).

## Body size and swimming speed

As before, we can run this model for the other two traits, to estimate the variance caused by the environments that individuals experience. For **body size**:

```
m_env_2<-asreml(fixed=trait_mee ~ 1 ,
                 random= ~ ped(ANIMAL) + giv(ANIMAL2),
```

```
                  data=Mermaids,
                  ginverse=list(ANIMAL=ainv,
                                ANIMAL2=p),
                  na.method.X="omit", na.method.Y="omit",
                  workspace=300e6,pworkspace=300e6,
                  maxiter=50)
```

```
summary(m_env_2)$varcomp
```

```
##                        gamma component  std.error   z.ratio constraint
## ped(ANIMAL)!ped  1.1940187  1.503473 0.15345829  9.797278   Positive
## giv(ANIMAL2).giv 0.8801978  1.108319 0.28918749  3.832529   Positive
## R!variance       1.0000000  1.259171 0.09400973 13.394049   Positive
```

We see an estimate of environmental variance that is close to the simulated variance of 1. However, the additive genetic and residual variance estimates remain above the simulated values of 1. Thus, this model does not account for all the variance (as the trait also includes maternal effect variance).

Similar overestimation of variances will be found for an equivalent model of **swimming speed**. Again, this is because there are other sources of variance in the trait which are not accounted for in this model.

```
m_env_3<-asreml(fixed=trait_mgee ~ 1 ,
                  random= ~ ped(ANIMAL) + giv(ANIMAL2),
                  data=Mermaids,
                  ginverse=list(ANIMAL=ainv,
                                ANIMAL2=p),
                  na.method.X="omit", na.method.Y="omit",
                  workspace=300e6,pworkspace=300e6,
                  maxiter=50)
```

In the section that follows, we show how parental effect variance can be included in these models.

# Parental effects

Parental effects occur when an individual's phenotype is partially determined by the environment provided by their parents. This could occur early in development, such as provisioning of micronutrients in eggs, or later, such as through post-natal food provisioning. Siblings raised together will typically experience similar parental effects, which can increase phenotypic similarity between relatives, beyond that caused by shared genetic effects. The effects that parents have on their own offspring can be driven by their own genes, or their own environments. Thus, we show here some different ways to include parental variance in the animal model. In all cases, maternal effects were simulated, and so maternal effect variances are estiamted in the models. Paternal effects, however, can also be estimated in the same way.

Both body size and swimming speed were simulated to have maternal effects on trait values. Specifically, body size is affected by maternal environmental effects, and swimming speed is affected by both maternal environmental and maternal genetic effects. We show first how maternal effect variance (with no assumption as to the cause of the maternal effect) can be included in the model, and then how specific maternal environmental and genetic variances can be estimated.

In the simplest case, maternal effect variance can be estiamted by including maternal identity as an additional random effect in the animal model. This accounts for variance shared by individuals that share a mother (full siblings and maternal half-siblings), above that which is due to additive genetic variance. The maternal effect is assumed to be normally distributed, with mean=0 and variance= $V_M$. There are no covariances between mothers, thus the variance-covariance matrix of this effect is the identity matrix ($I$).

To separate maternal effect variance from additive genetic variance, the data needs to have related individuals that do not share mothers (e.g. paternal half-siblings, cousins, or cross-fostered individuals). Equivalent models can be run for paternal effect variances (not shown in this tutorial), which requires there to be related individuals that do not share fathers (e.g. maternal half-siblings).

For **body size**, the model to estimate maternal variance is:

```
m_dam_2<-asreml(fixed=trait_mee ~ 1 ,
                random= ~   ped(ANIMAL) + giv(ANIMAL2) + dam,
                data=ped,
                ginverse=list(ANIMAL=ainv,
                             ANIMAL2=p),
                na.method.X="omit", na.method.Y="omit",
                workspace=300e6,pworkspace=300e6,
                maxiter=50)
```

As before, the *ped(ANIMAL)* term estimates additive genetic variance, and *giv(ANIMAL2)* the environmental variance. *dam* is the identity of the mother of each individual, so allows estimation of maternal variance.

Table S4: Variance estimates, and their proportions, for body size from an animal model that contains additive genetic (Va), environmental (Vn), maternal (Vm) and residual (Vr) variance. Statistical significance of random effects is calculated using log-likelihood tests

| Component | Estimate | Standard Error | z-ratio | pvalue | Proportion | Proportion SE |
|---|---|---|---|---|---|---|
| Va | 1.3142 | 0.19001 | 6.917 | <0.001 | 0.3369 | 0.04648 |
| Vn | 1.0788 | 0.26660 | 4.047 | <0.001 | 0.2765 | 0.05182 |
| Vm | 0.6532 | 0.09466 | 6.901 | <0.001 | 0.1674 | 0.02433 |
| Vr | 0.8551 | 0.10452 | 8.181 | | 0.2192 | 0.03554 |

This model estiamtes significant maternal variance (Vm), although this is less than the simulated maternal environmental variance of 1. Additive genetic variance is overestimated because not all maternal variance has been accounted for.

As the maternal effect is derived from the environment that mothers experience, and limited dispersal means that related mothers experience similar environments, related individuals experience similar maternal effects. Thus, we expect that mothers covary in their effects on offspring trait due to their shared environments, and we need to account for this covariance.

And for **swimming speed**:

```
m_dam_3<-asreml(fixed=trait_mgee ~ 1 ,
                random= ~   ped(ANIMAL) + giv(ANIMAL2) + dam,
                data=ped,
                ginverse=list(ANIMAL=ainv,
                             ANIMAL2=p),
                na.method.X="omit", na.method.Y="omit",
                workspace=300e6,pworkspace=300e6,
                maxiter=50)
```

Table S5: Variance estimates, and their proportions, for swimming speed from an animal model that contains additive genetic (Va), environmental (Vn), maternal (Vm) and residual (Vr) variance. Statistical significance of random effects is calculated using log-likelihood tests

| Component | Estimate | Standard Error | z-ratio | pvalue | Proportion | Proportion SE |
|---|---|---|---|---|---|---|
| Va | 1.4595 | 0.2220 | 6.573 | <0.001 | 0.3142 | 0.04495 |
| Vn | 1.0941 | 0.2707 | 4.041 | <0.001 | 0.2355 | 0.04647 |
| Vm | 1.3017 | 0.1462 | 8.904 | <0.001 | 0.2802 | 0.02961 |
| Vr | 0.7899 | 0.1167 | 6.766 | | 0.1701 | 0.03115 |

Again, there is an overesmtation of additive genetic variance. Maternal variance is estimated to be 1.3, but this underestimates the total amount of maternal variance, as there are both environmental and genetic maternal effects, each simulated with a variance of 1. As for body size, relatives have mothers that share environments, and so experience similar maternal environmental effects. Additionally, swimming speed includes a maternal genetic effect, so relatives have mothers that share genes affecting their maternal effects. Thus, covariance between mothers in their effects on offspring is caused both by environmental similarity and additive genetic relatedness. Although they are not simulated here, traits measured in real populations may have permanent maternal environment effects, in which case the maternal effect variance estimation above ($V_m$) should be retained.

## Maternal environmental variance

We can estimate maternal environmental variance in the same way that we estimated direct environmental variance. This makes use of the $S_n$ matrix, where each element describes the similarity of environment that two individual's experience. The maternal environmental variance is estimated with mean 0 and variance $V_{Mn}$, with the variance-covariance matrix determined by the $S_n$ matrix, $\mathbf{u_{Mn}} \sim N(0, \mathbf{S_n} * V_{Ma}$. Here, instead of considering the similarity in the trait between individuals $i$ and $j$ with environmental covariance $\mathbf{S_{n(i,j)}}$, we instead consider the similarity in the trait between offspring of mothers $k$ and $l$ with environmental covariance $\mathbf{S_{n(k,l)}}$.

For **body size**, the model with both direct and maternal environmental variance is:

```
m_menv_2<-asreml(fixed=trait_mee ~ 1 ,
                 random= ~   ped(ANIMAL) + giv(ANIMAL2) + giv(MOTHER2),
                 data=Mermaids,
                 ginverse=list(ANIMAL=ainv,
                               ANIMAL2=p,
                               MOTHER2=p),
                 na.method.X="omit", na.method.Y="omit",
                 workspace=300e6,pworkspace=300e6,
                 maxiter=50)
```

Note that this model uses around 15% CPU on the same computer described before, and takes 13 minutes to run.

For **swimming speed**:

```
m_menv_3<-asreml(fixed=trait_mgee ~ 1 ,
                 random= ~   ped(ANIMAL) + giv(ANIMAL2) + giv(MOTHER2),
                 data=Mermaids,
                 ginverse=list(ANIMAL=ainv,
                               ANIMAL2=p,
```

```
                              MOTHER2=p),
                 na.method.X="omit", na.method.Y="omit",
                 workspace=300e6,pworkspace=300e6,
                 maxiter=50)
```

## Maternal genetic variance

We know from the simulation of swimming speed that there is a maternal genetic effect upon this trait. Neglecting this effect causes overestimation of additive genetic variance (because relatives share both direct genetic effects, and can share maternal genetic effects that they experience). We therefore need to include maternal genetic variance in the model of this trait. In this case, we include a random effect with the variance-covariance of effects being determined by the additive genetic relatedness matrix ($\mathbf{A}$), as was done for the additive genetic variance. The difference here being that the variance is considered at the level of the mother, rather than at the level of the individual, such that for individual $i$ with mother $j$ the matrix is used to find the relatedness $A_{(j,k)}$, i.e. the relatedness between the mother $j$ and another individual $k$. Thus the random effect for maternal genetic variance is assumed to be normally distributed with a mean of zero and variance $\mathbf{A} * V_{Ma}$ (i.e. $\mathbf{u_{Ma}} \sim N(0, \mathbf{A} * V_{Ma})$).

To estimate maternal genetic variance in the model, we include the maternal identity ($MOTHER$) as a random effect, where this then has the variance-covariance structure determine by the inverse additive genetic relatedness matrix in the *ginverse* argument. Note that this model takes around 11 minutes to run on the same computer as before.

```
m_mgenetic_3<-asreml(fixed=trait_mgee ~ 1 ,
                 random= ~   ped(ANIMAL) + ped(MOTHER) + giv(ANIMAL2) + giv(MOTHER2),
                 data=Mermaids,
                 ginverse=list(ANIMAL=ainv,
                              MOTHER=ainv,
                              ANIMAL2=p,
                              MOTHER2=p),
                 na.method.X="omit", na.method.Y="omit",
                 workspace=300e6,pworkspace=300e6,
                 maxiter=50)
```

This shows that there is a considerable maternal genetic effect.

```
summary(m_mgenetic_3)$varcomp
```

```
##                       gamma component std.error  z.ratio constraint
## ped(ANIMAL)!ped   1.2135889 1.1190220 0.1934562 5.784367   Positive
## ped(MOTHER)!ped   0.8588372 0.7919137 0.1416314 5.591370   Positive
## giv(ANIMAL2).giv 1.2123289 1.1178602 0.2607856 4.286511   Positive
## giv(MOTHER2).giv 1.4587133 1.3450455 0.4107950 3.274250   Positive
## R!variance       1.0000000 0.9220767 0.1060360 8.695886   Positive
```

As a consequence of the inclusion of these effects, we can see that the heritability estimate ($V_a/(V_a + V_{Ma} + V_n + V_{Mn} + V_r)$) is:

```
nadiv:::pin(m_mgenetic_3, herit ~ V1 / ( V1 + V2 + V3 + V4 + V5))
```

```
##       Estimate         SE
## herit 0.211299 0.03819428
```

And the proportion of variance explained by the maternal genetic effect is:

```
nadiv:::pin(m_mgenetic_3, matgen ~ V2 / ( V1 + V2 + V3 + V4 +V5))
```

```
##         Estimate         SE
## matgen 0.1495329 0.02978131
```

Table S6: Variance estimates, and their proportions, for swimming speed from an animal model that contains additive genetic (Va), environmental (Vn), maternal (Vm) and residual (Vr) variance. Statistical significance of random effects is calculated using log-likelihood tests

| Component | Estimate | Standard Error | z-ratio | pvalue | Proportion | Proportion SE |
|---|---|---|---|---|---|---|
| Va | 1.1190 | 0.1935 | 5.784 | <0.001 | 0.2113 | 0.03819 |
| VMa | 0.7919 | 0.1416 | 5.591 | <0.001 | 0.1495 | 0.02978 |
| Vn | 1.1179 | 0.2608 | 4.287 | <0.001 | 0.2111 | 0.04261 |
| VMn | 1.3450 | 0.4108 | 3.274 | <0.001 | 0.2540 | 0.06149 |
| Vr | 0.9221 | 0.1060 | 8.696 | | 0.1741 | 0.02757 |

# Including epiallelic similarity

As well as environmental variables, we simulated epigenetic marks (CpG islands) for all individuals (described in the data simulation supplement). For each epigenetic island, the number of methylated CpG points varied between 0 and 20, with some determined by the environment an individual experienced.

As some of the epigenetic states varied according to the environments, they should account for variance in the phenotypic traits. We can therefore use them here to estimate (direct and maternal) epigenetic variance. We do this in a similar way to which we estimate environmental variance, with a matrix of epigenetic similarity between individuals.

In order to generate the epiallele similarity matrix, we first center and scale the epigenetic data, so that each island has a mean of 0 and variance of 1, as was done for environmental measures:

```
# mean centre, scale to have variance of 1
epl<-scale(epiallele, center=TRUE, scale=TRUE)
```

From the scaled epigenetic data, we generate the similarity matrix by estimating the Euclidean distance between all individuals. We then normalise values so that similar individuals have values closer to 1, and dissimilar have values closer to 0:

```
EP<-as.matrix(dist(epl, method="euclidean", diag=TRUE,upper=TRUE))
EP<-1-EP/(max(EP, na.rm=TRUE))

round(EP[1:10,1:10], digits=3)
```

```
##        ID1   ID2   ID3   ID4   ID5   ID6   ID7   ID8   ID9  ID10
## ID1  1.000 0.408 0.285 0.512 0.305 0.434 0.524 0.548 0.566 0.495
## ID2  0.408 1.000 0.481 0.491 0.404 0.370 0.571 0.513 0.318 0.379
## ID3  0.285 0.481 1.000 0.406 0.403 0.390 0.454 0.422 0.252 0.292
## ID4  0.512 0.491 0.406 1.000 0.449 0.445 0.593 0.599 0.419 0.566
## ID5  0.305 0.404 0.403 0.449 1.000 0.496 0.423 0.401 0.211 0.485
## ID6  0.434 0.370 0.390 0.445 0.496 1.000 0.418 0.472 0.403 0.526
## ID7  0.524 0.571 0.454 0.593 0.423 0.418 1.000 0.595 0.410 0.470
## ID8  0.548 0.513 0.422 0.599 0.401 0.472 0.595 1.000 0.488 0.532
## ID9  0.566 0.318 0.252 0.419 0.211 0.403 0.410 0.488 1.000 0.447
## ID10 0.495 0.379 0.292 0.566 0.485 0.526 0.470 0.532 0.447 1.000
```

```r
# The matrix inverse is then taken for use in the model.
EP<-as(solve(EP),"dgCMatrix")
EP<-as.matrix(EP)
```

We can then include the model of epigenetic similarity between individuals, which accounts for its effects on the direct and parental environmental effects. For **tail-fin colour** we estimate the additive genetic variance and the direct epigenetic variance:

```r
m_epiallele_1<-asreml(fixed=trait_ae ~ 1 ,
                   random= ~   ped(ANIMAL) + giv(ANIMAL2),
                   data=Mermaids,
                   ginverse=list(ANIMAL=ainv,
                                 ANIMAL2=EP),
                   na.method.X="omit", na.method.Y="omit",
                   workspace=300e6,pworkspace=300e6,
                   maxiter=50)
```

The results from this model then show that the epigenetic similarity explains a significant amount of variance.

```r
summary(m_epiallele_1)$varcomp
```

```
##                       gamma component  std.error  z.ratio constraint
## ped(ANIMAL)!ped  1.603789 1.0968468 0.12352602 8.879480   Positive
## giv(ANIMAL2).giv 1.458992 0.9978189 0.17843582 5.592032   Positive
## R!variance       1.000000 0.6839097 0.08559452 7.990111   Positive
```

Again, we can show the proportions of variance that are estimated for additive genetic variance:

```
##        Estimate       SE
## herit 0.3947515 0.039955
```

and epigenetic variance:

```
##         Estimate         SE
## direpi 0.3591117 0.04853229
```

Table S7: Variance estimates, and their proportions, for swimming speed from an animal model that contains additive genetic (Va), epigenetic (Vepi), and residual (Vr) variance. Statistical significance of random effects is calculated using log-likelihood tests

| Component | Estimate | Standard Error | z-ratio | pvalue | Proportion | Proportion SE |
|---|---|---|---|---|---|---|
| Va | 1.0968 | 0.12353 | 8.879 | <0.001 | 0.3948 | 0.03995 |
| Vepi | 0.9978 | 0.17844 | 5.592 | <0.001 | 0.3591 | 0.04853 |
| Vr | 0.6839 | 0.08559 | 7.990 | | 0.2461 | 0.04006 |

As with the environmental variance, we can also estimate maternal epigenetic variance, using **body size**

```r
m_epiallele_2<-asreml(fixed=trait_mee ~ 1 ,
                   random= ~   ped(ANIMAL) + giv(ANIMAL2) + giv(MOTHER2),
                   data=Mermaids,
                   ginverse=list(ANIMAL=ainv,
                                 ANIMAL2=EP,
                                 MOTHER2=EP),
                   na.method.X="omit", na.method.Y="omit",
                   workspace=300e6,pworkspace=300e6,
                   maxiter=50)
```

```r
summary(m_epiallele_2)$varcomp
```

```
##                       gamma component std.error  z.ratio constraint
## ped(ANIMAL)!ped  1.778090 1.0914441 0.1568775 6.957303   Positive
## giv(ANIMAL2).giv 1.767338 1.0848442 0.2009161 5.399488   Positive
## giv(MOTHER2).giv 1.139276 0.6993208 0.1201258 5.821568   Positive
## R!variance       1.000000 0.6138294 0.1022610 6.002575   Positive
```

The epigenetic similarity matrix estimates significant direct epigenetic variance in both traits, and maternal epigenetic variance for body size, although this underestimates true maternal variance.

## Including a social network

We simulated a social network wherein individuals had connections within generations and between adjacent generations. Connections between generations were more likely for individuals that were close in space, and decreased with increasing distance. Between adjacent generations there was a higher probability of connections between mothers and offspring than for other pairs of individuals, again with decreasing probability of connections with spatial distance.

We can visualise the social network in 3D space, in a similar way to the pedigree. The physical locations of individuals are plotted on the X and Y axes (locations on spatial grid), and generations on the Z axis. Individual points are coloured by sex (blue for males, red for females). Each line represents an (unweighted) connection in the social network. Running the code below will produce a moveable 3D plot. We show the equivalent plot for just the first 3 generations below.

```r
social2<-social
social2[upper.tri(social2)]<-NA

social2<-setNames(melt(social2, na.rm=TRUE), c('ind.1', 'ind.2', 'values'))
social2<-social2[which(social2$values!=0),]



colors <- Mermaids$sex
colors[which(colors==1)]<- "red"
colors[which(colors==0)]<- "blue"


plot3d(Mermaids$Xloc,Mermaids$Yloc,Mermaids$generation,
       type="p", size=5, col=colors,
       xlab="X", ylab="Y", zlab="Generation")


for(i in 1:nrow(social2)){

  id1<-social2$ind.1[i]
  id2<-social2$ind.2[i]

  x1<-Mermaids$Xloc[which(Mermaids$id==id1)]
  x2<-Mermaids$Xloc[which(Mermaids$id==id2)]

  y1<-Mermaids$Yloc[which(Mermaids$id==id1)]
  y2<-Mermaids$Yloc[which(Mermaids$id==id2)]
```

```
  z1<-Mermaids$generation[which(Mermaids$id==id1)]
  z2<-Mermaids$generation[which(Mermaids$id==id2)]


  lines3d(z= c(z1,z2), y = c(y1,y2), x = c(x1,x2), col = "grey85", lwd =1)
  }
}
```

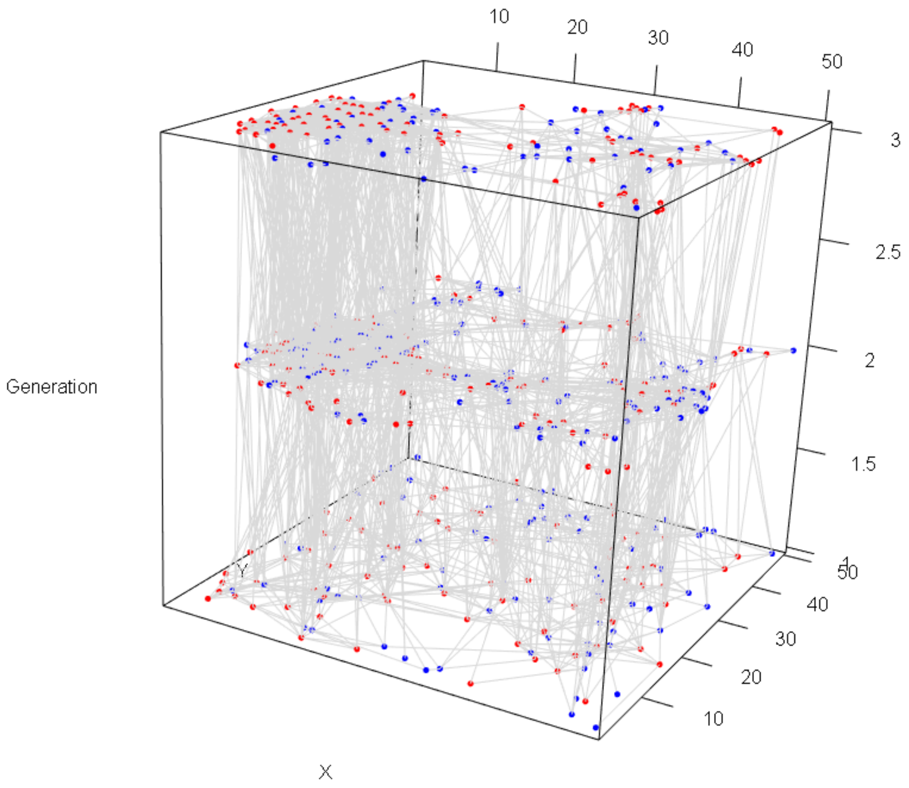


Here, we show how social network links between individuals can be converted to a matrix of distances between individuals in the network, and passed to the model to account for social structure and possible cultural inheritance. Initially, we carry this out using unweighted distances between individuals: individuals that have a direct connection have 1 in the matrix, and all other individuals have 0.

```
### Go from weighted to unweighted distances
social2<-social
social2[which(social2>0)]<-1
```

From this, we then want to find the geodesic distances between individuals. This is the length of the shortest path between individuals in the matrix, such that directly connected individuals have a path length of 1, individuals with one intermediate connection get a path length of 2, and so on

```r
# gets geodesic distances between individuals
# (ie number of steps on the matrix to connect them)
net<-graph.adjacency(social2, mode = c( "undirected"), weighted = NULL, diag=FALSE)
net2<-distances(net)

net2[1:10,1:10]
```

As we want individuals to be connected to themselves, we add 1 to every element of the matrix, so that the shortest paths are from an individual to itself.

We then normalise the matrix so that values lie between 1 and 0, which gives individual proximities between two individuals, with 1 being the closest proximity and 0 the furthest.

```r
# add 1 so individuals are connected most strongly to themselves

net3<-net2+1

# scale matrix between 0 and 1
net4<-1/net3
```

We then take the matrix inverse, as with the other matrices.

```r
# inverse
N<-as(solve(net4),"dgCMatrix")
N<-as.matrix(N)
```

This matrix can then be passed to the model to estimate the variance associated with the social network connections between individuals. For **tail-fin colour**:

```r
m_social_1<-asreml(fixed=trait_ae ~ 1 ,
                   random= ~   ped(ANIMAL) + giv(ANIMAL2) ,
                   data=Mermaids,
                   ginverse=list(ANIMAL=ainv,
                                 ANIMAL2=N),
                   na.method.X="omit", na.method.Y="omit",
                   workspace=300e6,pworkspace=300e6,
                   maxiter=50)
```

The results from this model show that there is a small but significant amount of variance explained by the direct social network effects, but it does not account for all the variance, so the additive genetic and residual variances remain overestimated.

```r
summary(m_social_1)$varcomp
```

```
##                       gamma component std.error   z.ratio constraint
## ped(ANIMAL)!ped  1.2250548 1.5014729 0.1799399  8.344304   Positive
## giv(ANIMAL2).giv 0.4004846 0.4908489 0.0812790  6.039062   Positive
## R!variance       1.0000000 1.2256373 0.1067701 11.479221   Positive
```

Table S8: Variance estimates, and their proportions, for swimming speed from an animal model that contains additive genetic (Va), social (Vc), and residual (Vr) variance. Statistical significance of random effects is calculated using log-likelihood tests

| Component | Estimate | Standard Error | z-ratio | pvalue | Proportion | Proportion SE |
|---|---|---|---|---|---|---|
| Va | 1.5015 | 0.17994 | 8.344 | <0.001 | 0.4666 | 0.04368 |
| Vc | 0.4908 | 0.08128 | 6.039 | <0.001 | 0.1525 | 0.02352 |
| Vr | 1.2256 | 0.10677 | 11.479 | | 0.3809 | 0.04041 |

| Component | Estimate | Standard Error | z-ratio | pvalue | Proportion | Proportion SE |
|---|---|---|---|---|---|---|

For **body size**, we can also estimate maternal social network variance

```
m_social_2<-asreml(fixed=trait_mee ~ 1 ,
                   random= ~   ped(ANIMAL) + giv(ANIMAL2) + giv(MOTHER2),
                   data=Mermaids,
                   ginverse=list(ANIMAL=ainv,
                                 ANIMAL2=N,
                                 MOTHER2=N),
                   na.method.X="omit", na.method.Y="omit",
                   workspace=300e6,pworkspace=300e6,
                   maxiter=50)
```

```
summary(m_social_2)$varcomp
```

```
##                        gamma component std.error   z.ratio constraint
## ped(ANIMAL)!ped  0.8129438 1.2128913 0.1996608  6.074760   Positive
## giv(ANIMAL2).giv 0.1773093 0.2645409 0.0894787  2.956469   Positive
## giv(MOTHER2).giv 0.4169444 0.6220703 0.1263996  4.921456   Positive
## R!variance       1.0000000 1.4919744 0.1271384 11.735041   Positive
```

Again, whilst this estimates significant social network variance for both direct and maternal cases, these underestimate the true environment variance. This is because of the way the social network was simulated, compared to the actual simulations structure, which was based on environmental variables.

## Including a weighted social network

In the example shown above, we reduced the social network to a binary state; individuals were either linked directly or not, and from that we calculated path lengths. Researchers may want instead to include weighted paths, for example with numbers representing the number of times a pair of individuals was recorded interacting.

In this case, we calculate the geodesic distances between individuals using weighted path lengths. This is done using the $distance_w$ function from the package *tnet*.

```
#changes to tnet standard network
net2<-as.tnet(social)

# Finds shortest path lengths between each pair, using weighting information
net3<-distance_w(net2)

# One individual is actually missing from this (ID642)
# as it has no social network connections.
# We add this back to the matrix later

# have to slightly reorder row/column names to match the fact one is missing
names<-c(as.character(Mermaids$id[which(Mermaids$id!="ID642")]))

colnames(net3)<-rownames(net3)<-names

# This initially has NAs on the diagonal,
# but we need individuals to be most strongly connected to themselves
# Add values so shortest path lengths are individuals to themselves
```

```r
diag(net3)<-0

# Scale the matrix so that short paths have values close to 1,
# and long paths values close to 0
net3<-1-net3/max(net3, na.rm=TRUE)

# One individual is actually missing from this (ID642)
# as it has no social network connections.
# We can add the missing individual back with connections of 0

net3<-cbind(net3, rep(0, nrow(net3)))
net3<-rbind(net3, rep(0, ncol(net3)))

colnames(net3)<-rownames(net3)<-c(names, "ID642")

# diagonals must be 1
diag(net3)<-1

# Gets the matrix inverse, as before
Nw<-as(solve(net3),"dgCMatrix")
Nw<-as.matrix(Nw)
```

The matrix of weighted network connections is included in the same way as the simple social network to account for the variance in the model caused by the social network

```r
m_social_weighted<-asreml(fixed=trait_ae ~ 1 ,
                 random= ~   ped(ANIMAL) + giv(ANIMAL2) ,
                 data=Mermaids,
                 ginverse=list(ANIMAL=ainv,
                               ANIMAL2=Nw),
                 na.method.X="omit", na.method.Y="omit",
                 workspace=300e6,pworkspace=300e6,
                 maxiter=50)

saveRDS(m_social_weighted, file=paste(savemodelpath3, "model_ae_weighted.RDS", sep=""))
```

In this case, the estimated variances are smaller than those generated by the unweighted matrix.

```r
summary(m_social_weighted)$varcomp
```

```
##                        gamma component  std.error   z.ratio constraint
## ped(ANIMAL)!ped  1.1237425 1.6421046 0.18593303  8.831699   Positive
## giv(ANIMAL2).giv 0.1331685 0.1945968 0.06945889  2.801611   Positive
## R!variance       1.0000000 1.4612820 0.10934371 13.364116   Positive
```

# Plotting the models

We can visualise the variance explained by different models in each of the parameters in a plot. This is done by first combining all the variances, and their proportions estimated using nadiv:::pin, for each model into one data frame. This is shown here only for **tail-fin colour** :

```r
head(plotdat)
```

```
##   Trait                         Model component       est          SE   z_ratio
```

```
## 1     1             Genetic only        Va 1.7135746 0.18766455  9.131051
## 2     1             Genetic only        Vr 1.4721378 0.10966438 13.424029
## 3     1 Environmental Similarity        Va 1.1003540 0.12400652  8.873356
## 4     1 Environmental Similarity        Vn 0.9860696 0.21690876  4.546011
## 5     1 Environmental Similarity        Vr 0.8940308 0.07390975 12.096249
## 6     1    Epigenetic Similarity        Va 1.0968468 0.12352602  8.879480
##        p  prop.est    prop.SE
## 1 <0.001 0.5378937 0.04249887
## 2   <NA> 0.4621063 0.04249887
## 3 <0.001 0.3691900 0.04191195
## 4 <0.001 0.3308454 0.05125380
## 5   <NA> 0.2999646 0.03708500
## 6 <0.001 0.3947515 0.03995500
```

```r
# Need the upper and lower standard errors for error bars

plotdat$upper<-plotdat$est + plotdat$SE
plotdat$lower<-plotdat$est - plotdat$SE

# gets all the different components into the right order for plotting
plotdat$biglab<-as.factor(paste(plotdat$Model,plotdat$component,sep="."))

# need to be ordered in reverse from how they will be plotted
# ie. first level is plotted at the bottom of the y axis
# last level is plotted at the top
plotdat$biglab <- factor(plotdat$biglab,
                         levels(plotdat$biglab)[c(11,10,9,
                                                  6,5,4,
                                                  3,2,1,
                                                  8,7)])


small.labels=c(expression(italic("a")),
               expression(italic("r")),
               expression(italic("a")),
               expression(italic("n")),
               expression(italic("r")),
               expression(italic("a")),
               expression(italic("epi")),
               expression(italic("r")),
               expression(italic("a")),
               expression(italic("c")),
               expression(italic("r")))

# select colours

pal<-c("#bc3d36", "#eebca1", "#8898b1")

# associate labels for colours

plotdat$col<-"c"
plotdat$col[which(plotdat$component=="Va")]<-"a"
plotdat$col[which(plotdat$component=="Vn"|
                    plotdat$component=="Vepi"|
```

```
                    plotdat$component=="Vc")]<-"b"
plotdat$col<-as.factor(plotdat$col)


modellabs<-c("Additive Genetic",
             "Environmental Similarity",
             "Epigenetic Similarity",
             "Cultural Similarity")
modelx<-rep(2.5,4)

modely<-c(11,9,6,3)
```

We then use ggplot2 to produce a plot of all the models for tail-fin colour:

```
ggplot(plotdat,aes(x=est,y=biglab, colour=col)) +
  theme(axis.line = element_line(colour = "black", size=1, lineend="butt"),
        panel.grid.major = element_blank(),
        panel.grid.minor = element_blank(),
        panel.border = element_rect(colour = "black", fill=NA, size=1.5),
        axis.text=element_text(size=15, colour="black"),
        axis.title.x=element_text(size=15, vjust=0),
        axis.title.y=element_text(size=15, vjust=1.5),
        axis.ticks = element_line(colour = "black", size=1),
        panel.background = element_blank(),
        legend.title = element_text(size=10, face="bold"),
        legend.text = element_text(size=10),
        legend.position = "bottom") +
  geom_point(size=5, shape=19) +
  geom_errorbarh(aes(xmax = upper, xmin = lower), height = 0.3, size=0.6) +
  #scale_x_continuous(breaks = seq(0,1.5,0.25), labels = seq(0,1.5,0.25)) +
  scale_y_discrete(labels=rev(small.labels)) +
  geom_abline(intercept=3.5, slope=0, linetype=2) +
  geom_abline(intercept=6.5, slope=0, linetype=2) +
  geom_abline(intercept=9.5, slope=0, linetype=2) +
  geom_vline(xintercept=1, linetype=2) +
  labs(x="Variance", y="") +
  scale_colour_manual(values = pal,
                      name="Variance Component",
                      breaks=c("a","b","c"),
                      labels=c("Additive Genetic",
                               "Direct Similarity Matrix",
                               "Residual")) +
   annotate("text", x = modelx, y = modely, label = modellabs, size=5, hjust=1)
```

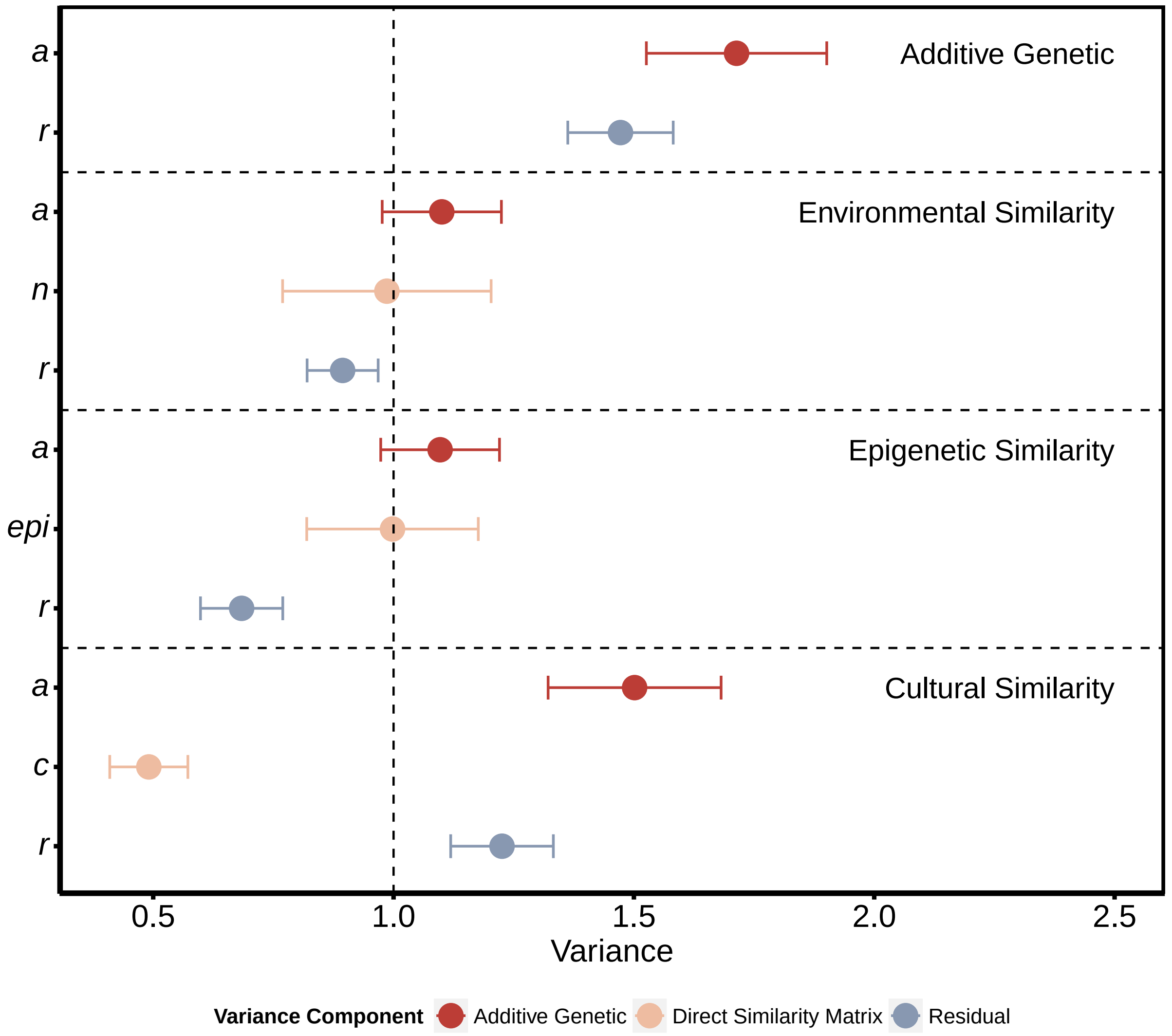


We can also plot the proportions. This requires functions from the R library *scales*:

```
# Need to reorder levels so they plot in the right order
plotdat$Model <- factor(plotdat$Model,
                         levels(plotdat$Model)[c(4,2,1,3)])
```

```
ggplot(plotdat,aes(x = Model, y = est, fill=col)) +
  geom_bar(position = "fill",stat = "identity",colour="black", size=1) +
  scale_y_continuous(labels = percent_format())+
  theme(axis.line.x = element_blank(),
        axis.line.y = element_blank(),
        panel.grid.major = element_blank(),
        panel.grid.minor = element_blank(),
        panel.border = element_blank(),
        panel.background = element_blank(),
        legend.title = element_text(size=18, face="bold"),
        legend.text = element_text(size=15),
        legend.position = "none") +
  scale_fill_manual(values = pal)+
  coord_flip()+
```

```r
  labs(y="Proportion of Variance", x="Model")
```

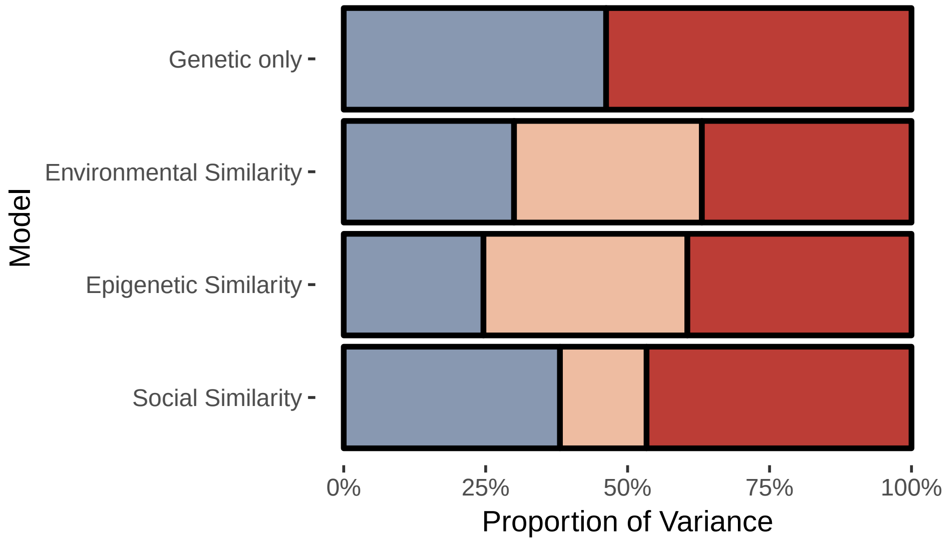


## Using a genomic relatedness matrix

Some readers may wish to use a genomic relatedness matrix (GRM), such as can be created using SNP chips, rather than a pedigree. These can be accomodated in such models easily. We have not simulated one here, but give code that could be used if one exists. Where users have a GRM matrix, it should be a matrix of values between 1 and 0 that give the proportion of genome (e.g. SNPs) shared between each pair of individuals.

```r
# read in matrix from file
GRM<-read.table(file="GRM.txt",header=TRUE)

# make sure that column and row names match
# (should be individual names/ids)
colnames(GRM)<-rownames(GRM)


GRM<-as.matrix(GRM)


m_basic_GRM<-asreml(fixed=trait_ae ~ 1 ,
                   random= ~   ped(ANIMAL) ,
                   data=Mermaids,
                   ginverse=list(ANIMAL=GRM),
                   na.method.X="omit", na.method.Y="omit")
```

## Running models in MCMCglmm

Some readers may wish to use MCMCglmm, a freely available software for running Bayesian mixed models, to run these models. Generally, these models have a similar set up to those shown above, with a few differences in the way that the pedigree and similarity matrices are prepared for the models.

It should be noted that these models are considerably slower to run in MCMCglmm than in ASreml-R. Due to this time constraint, we would recommend that researchers consider carefully whether they wish to use MCMCglmm or ASreml-R. For the models including a non-genetic similarity matrix we found that 1000 iterations took around 24 hours to run on a windows computer with 32GB of RAM and 3.40GHz CPU. Evidently, a more powerful computer would reduce this time to some extent.

As such, we include a minimal tutorial here, with models run for only a small number of iterations, to demonstrate how to utilise such models. To carry out a complete analysis using this tutorial, however, we recommend increasing the number of iterations, the burn-in period, and the thinning interval used. This is done by changing the arguments *nitt*, *burnin*, and *thin* in the model codes. MCMCglmm has a default of 13000, 3000, and 10 for each of these measures. We recommend increasing these considerably to carry out robust analyses.

MCMCglmm requires priors to be passed to models. We do not intend this tutorial to be a complete guide to priors, and we include simple priors for the models here. For those who are interested, we recommend reading Hadfield (2016) and Villemereuil (2012).

## Additive genetic relatedness

In order to run the model, we then must take the inverse of the relatedness matrix between individuals. Note that this uses a different function than the equivalent function for models run in ASreml-r.

```
# gets pedigree inverse for the model

# first get the pedigree from the data
pedigree<-Mermaids[,c("id","dam","sire")]

# inserts any missing individuals into the pedigree

missing<-unique(Mermaids$id[which(!Mermaids$id%in%pedigree[,1])])
if(length(missing)>0){
  pedigree<-insertPed(pedigree, missing)
}

# Orders the pedigree so that parents come before their offspring
pedigree<-orderPed(pedigree)

# Gets the inverse of the relatedness matrix
Ainv<-inverseA(pedigree)$Ainv
```

## Basic model

We show first a basic animal model, which includes only the additive genetic variance, and residual variance, on the trait. Here, we fit only the mean/intercept of the reponse variable, by calling *trait_ae ~ 1* , and fit the single random effect of the pedigree with *random= ~ ANIMAL*. This is linked to the inverse relatedness matrix with the ginverse argument (*ginverse=list(ANIMAL=ainv)*).

```
# Basic model - includes only additive genetic variance

prior1 <- list(G = list(G1 = list(V = 1, nu = 0.002)),
                 R = list(V = 1, nu = 0.002))

MCMC_basic<-MCMCglmm(trait_ae ~ 1 ,
                 random= ~   ANIMAL ,
```

```
                 data=Mermaids,
                 ginverse=list(ANIMAL=Ainv),
                 prior=prior1)
```

We can see the estimated variance components by calling:

```
summary(MCMC_basic)
```

```
##
##  Iterations = 3001:12991
##  Thinning interval  = 10
##  Sample size  = 1000
##
##  DIC: 6528.186
##
##  G-structure:  ~ANIMAL
##
##        post.mean l-95% CI u-95% CI eff.samp
## ANIMAL     1.705    1.357    2.066    428.8
##
##  R-structure:  ~units
##
##       post.mean l-95% CI u-95% CI eff.samp
## units     1.489    1.286    1.701    525.4
##
##  Location effects: trait_ae ~ 1
##
##             post.mean l-95% CI u-95% CI eff.samp pMCMC
## (Intercept)  -0.04969 -0.30000  0.21770     1000 0.664
```

The values given under G-structure in the model summary are the values for the random effects (in this case additive genetic variance, the *ANIMAL* term) and R-structure gives the residual variance estimates (*units*). The posterior means of estimates (*post.mean*) are given, along with the lower (*l-95% CI*) and upper (*u-95% CI*) limits of the 95% confidence intervals for these estimates. *eff.samp* shows the effective sample size for each parameter. Ideally these should be over 1000, thus the values for the random and residual variance estimates are low. This can be improved by running the model for a longer period of time, and increasing the thinning interval to reduce autocorrelation. This is done by altering the *nitt* (number of iterations) argument, *burnin* (the burn-in period, which is discarded) and *thin* (the thinning interval - how often iterations are sampled). Be aware that this means that the model runs for a longer period of time:

```
# Basic model - includes only additive genetic variance

prior1 <- list(G = list(G1 = list(V = 1, nu = 0.002)),
                 R = list(V = 1, nu = 0.002))

MCMC_basic2<-MCMCglmm(trait_ae ~ 1 ,
                 random= ~   ANIMAL ,
                 data=Mermaids,
                 ginverse=list(ANIMAL=Ainv),
                 prior=prior1,
                 nitt=13000*10,
                 thin=10*10,
                 burnin=3000*10)
```

This gives effective sample sizes that are much larger, although there is not much change to the vraiance estimates.

```r
summary(MCMC_basic2)
```

```
##
##  Iterations = 30001:129901
##  Thinning interval  = 100
##  Sample size  = 1000
##
##  DIC: 6523.932
##
##  G-structure:  ~ANIMAL
##
##        post.mean l-95% CI u-95% CI eff.samp
## ANIMAL     1.726    1.386    2.098     1000
##
##  R-structure:  ~units
##
##       post.mean l-95% CI u-95% CI eff.samp
## units     1.465    1.249    1.669     1000
##
##  Location effects: trait_ae ~ 1
##
##             post.mean l-95% CI u-95% CI eff.samp pMCMC
## (Intercept)  -0.04504 -0.29459  0.21860     1000 0.738
```

This model has mixed reasonably well, which can be seen by plotting the traces:

```r
plot(MCMC_basic2)
```

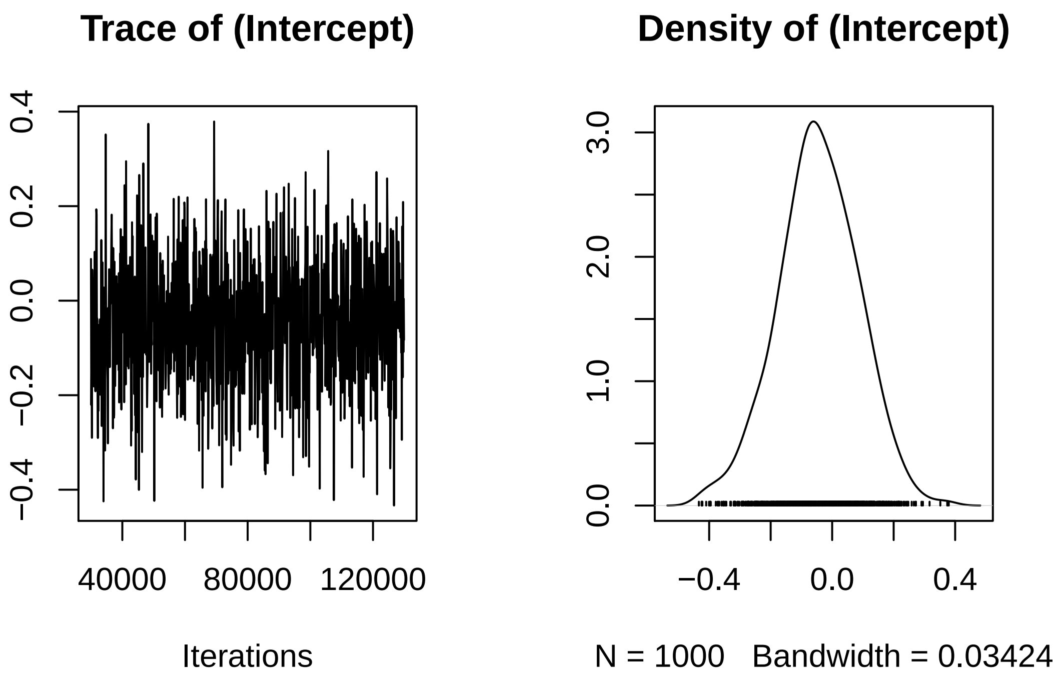

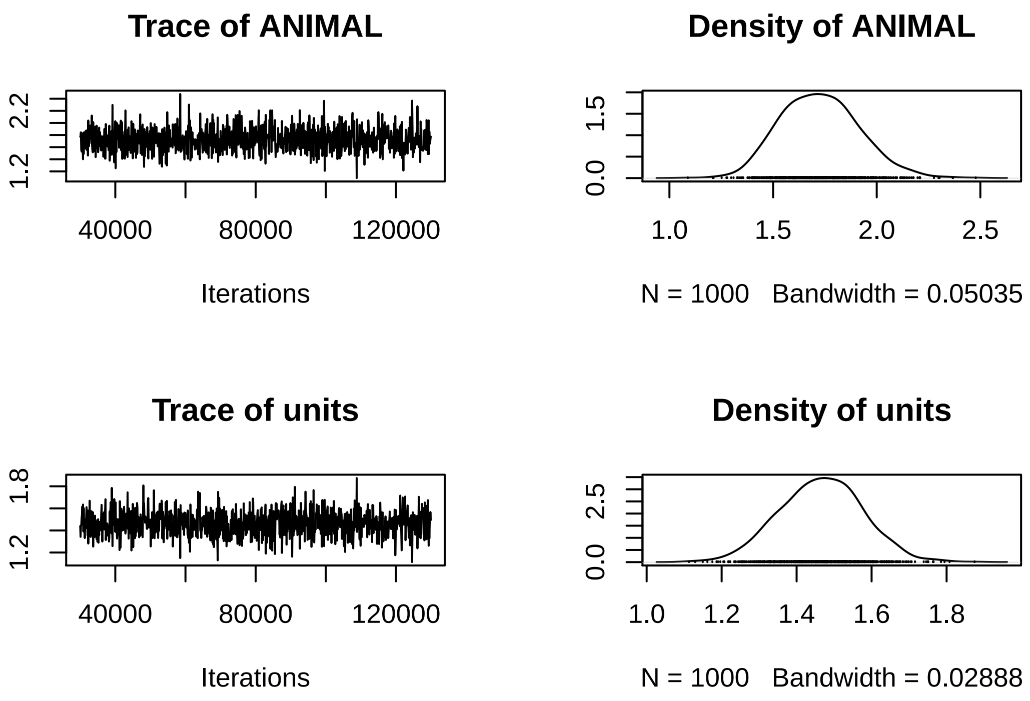


### Including environmental similarity

In order to account for non-genetic similarity between individuals, we can fit an additional random effect that account for variance in tail-fin colour. We show how this can be done using continuous environmental measures, as above.

As with the matrix shown for the ASreml-R models, we first scale and centre the data, so that the mean is 0 and variance is 1 for each of the environmental measures. The Euclidean distances between individuals are then taken, and values normalised to be between 0 and 1, just as above.

```
# First gets the relevant columns from the data
enm<-Mermaids[,c("e_var_1", "e_var_2", "e_var_3", "e_var_4", "e_var_5")]

# Centers the data, and scales by standard deviation
enm<-scale(enm, center=TRUE, scale=TRUE)

rownames(enm)<-Mermaids$id

# similarity matrix between individuals
# gets the euclidean distance between each individual's environment parameters

p<-as.matrix(dist(enm, method="euclidean", diag=TRUE,upper=TRUE))


# then scales so that the values are between 0 and 1
p<-1-p/max(p, na.rm=TRUE)
```

We then need to pass the matrix inverse to MCMCglmm, so it is inverted using the *solve()* function. This is the same as the way we generate the matrix for ASreml-R, but we do not need the additional step of

changing back into a matrix (*as.matrix(p)* argument is included after this step for ASreml-R only).

```
# gets the matrix inverse
p<-as(solve(p),"dgCMatrix")
```

The full model that includes additive genetic and environmental variance is then run with the following code. The environmental similarity matrix is now passed as an additional argument to the *ginverse*, and connected to the phenotypes using the *giv(ANIMAL2)*.

Before starting this model, it should be noted that this is considerably slower than the models above - this took over 7 hours to run on a computer using 32GB of RAM and 3.40GHz CPU. The model below is run with the default number of iterations, thinning interval, and burn-in period, but o give reliable estimates these parameters should be increased further, which will increase the running time.

```
# Environmental similarity model

prior2 <- list(G = list(G1 = list(V = 1, nu = 0.002),
G2 = list(V = 1, nu = 0.002)),
                 R = list(V = 1, nu = 0.002))

MCMC_env<-MCMCglmm(trait_ae ~ 1 ,
                 random= ~   ANIMAL + ANIMAL2 ,
                 data=Mermaids,
                 ginverse=list(ANIMAL=Ainv,
                               ANIMAL2=p),
                 prior=prior2)
```

The variance estimates are now both around 1 (*ANIMAL* is additive genetic variance as before, *ANIMAL2* is environmental variance):

```
summary(MCMC_env)
```

```
##
##  Iterations = 3001:12991
##  Thinning interval  = 10
##  Sample size  = 1000
##
##  DIC: 5719.826
##
##  G-structure:  ~ANIMAL
##
##        post.mean l-95% CI u-95% CI eff.samp
## ANIMAL     1.093   0.8643    1.323    511.8
##
##                ~ANIMAL2
##
##         post.mean l-95% CI u-95% CI eff.samp
## ANIMAL2    0.9981   0.6756    1.391    170.1
##
##  R-structure:  ~units
##
##       post.mean l-95% CI u-95% CI eff.samp
## units     0.901   0.7589    1.032    486.9
##
##  Location effects: trait_ae ~ 1
##
##             post.mean l-95% CI u-95% CI eff.samp pMCMC
```

```
## (Intercept)    0.1370  -0.9933   1.2381     1000 0.818
```

Nevertheless, the effective sample sizes are small, particularly for the environmental variance (*ANIMAL2*). Thus, the *nitt*, *thin* and *burnin* values should be increased. We do not present that here due to time constraints (for example, increasing the iterations by 10 as shown above will increase the run time to around 70 hours).

## Including parental environmental variance

Finally, maternal environmal variance can also be included in the model, in the same way as the ASreml models shown above. We run this model only for 1000 iterations sing this took around 1.5 hours. In reality this model should be run much longer (likely upwards of 130000 iterations).

```r
# Environmental similarity model

prior3 <- list(G = list(G1 = list(V = 1, nu = 0.002),
G2 = list(V = 1, nu = 0.002),
G3 = list(V = 1, nu = 0.002)),
                R = list(V = 1, nu = 0.002))

MCMC_matenv<-MCMCglmm(trait_mee ~ 1 ,
                random= ~   ANIMAL + ANIMAL2 + MOTHER2,
                data=Mermaids,
                ginverse=list(ANIMAL=Ainv,
                              ANIMAL2=p,
                              MOTHER2=p),
                prior=prior3,
                nitt=1000,
                thin=1,
                burnin=30)
```

```r
summary(MCMC_matenv)
```

```
##
##  Iterations = 31:1000
##  Thinning interval  = 1
##  Sample size  = 970
##
##  DIC: 5734.809
##
##  G-structure:  ~ANIMAL
##
##        post.mean l-95% CI u-95% CI eff.samp
## ANIMAL     1.259   0.9836      1.5     43.7
##
##                 ~ANIMAL2
##
##         post.mean l-95% CI u-95% CI eff.samp
## ANIMAL2      1.13   0.7537    1.506    19.04
##
##                 ~MOTHER2
##
##         post.mean l-95% CI u-95% CI eff.samp
## MOTHER2    0.9497   0.6512    1.382    13.09
##
##  R-structure:  ~units
```

```
##
##       post.mean l-95% CI u-95% CI eff.samp
## units    0.8536   0.7077    1.031    37.48
##
##  Location effects: trait_mee ~ 1
##
##             post.mean l-95% CI u-95% CI eff.samp pMCMC
## (Intercept)   -0.1716  -1.2388   1.0941    674.7 0.781
```

# References


Hadfield, J. (2016). MCMCglmm Course Notes.

Villemereuil, P. de. (2012). Estimation of a biological trait heritability using the animal model: how to use the MCMCglmm R package. 36.
