# Supplementary material for: A guide to using a multiple-matrix animal model to disentangle genetic and nongenetic causes of phenotypic variance
Source: PLoS One. 2018 Oct 12;13(10):e0197720. doi: 10.1371/journal.pone.0197720 (PMC6193571; doi:10.1371/journal.pone.0197720)
Supplement: S2 File — Thorough detailing on the simulation used to create the data in this paper. (HTML) [file pone.0197720.s002.html]

Data Simulation


# Data Simulation

- Pedigree and dispersal
- Environment
- Phenotypic Traits
  - Additive genetic effects
  - Environmental effects
  - Maternal effects
  - Residual variance
  - Phenotypic trait values
- Epi-alleles
- Social Network

Data was simulated for an semelparous mermaid population that lives in a “lagoon” of a 50x50 spatial grid. The environment varies across the grid, with spatial autocorrelation, as determined by five environmental variables.

We here provide documentation of how this was simulated, with complete code and functions, for those who wish to recreate the simulation or vary the parameters. Libraries used were:

```
library(raster)
library(lattice)
library(mvnfast)
library(pedantics)
library(sequoia)
library(ape)
library(synbreed)
library(asreml)
library(reshape2)
library(igraph)
```

# Pedigree and dispersal

The population is simulated across 10 semelparous (non-overlapping) generations. Each generation has a population size randomly varying between 150 and 250 individuals (Figure ). Individuals are randomly assigned to be either male or female (no hermaphroditism).

Within a generation, ~50% of the population is considered to be breeding (in mating pairs), and ~50% non-breeding. Within the randomly selected breeding sub-population, males and females are assigned to breeding pairs at random. Each offspring from the next generation is then assigned to a parental pair at random, allowing variation in the number of offspring in each family group. Extra-pair mating is simulated at a rate of 10%. For the 10% that are extra-pair, one parent is resampled from the breeding populations, with equal likelihood of resampling either the mother or father. In this way, the population contains a mixture of full- and half-siblings. The pedigree is shown in Figure .

Individuals live in the 50x50 spatial grid that repesents the lagoon. The first generation of individuals are assigned random positions in the lagoon. After the first generation, individuals disperse away from their mothers. The distance of dispersal is determined by a log-normal function, with mean=1 and variance=1. The angle of dispersal is sampled at random between 1 and 360. Due to the nature of this dispersal function, some individuals disperse off the edge of the grid. In this case, these offspring are considered to have dispersed out of the suitable habitat and or died. As such, they are not considered potential parents for the next generations, and are removed entirely from the dataset. The final population size for each generation is therefore slightly smaller than the initial simulated population size.

The code used to similate the pedigree and dispersal is:

```
##################################
####### PEDIGREE FUNCTION ########
##################################

# function to create a pedigree with dispersal
# inputs:
# nids = list of number of individuals per generation
# ngenerations = number of generations to simulate
# epm = rate of extra-pair mating (defaults to NULL, no extra-pair)
# missing = probability that one parent is missing in the pedigree
# nonb = proportion of each generation that is non-breeding
# gridsize = length of one size of (square) spatial grid 
# dispmean = mean dispersal distance (lognormal)
# dispvar = variance in dispersal distance (lognormal)


pedfun<-function(nids, ngenerations, epm=NULL, missing=NULL, nonb=0.4, 
                 gridsize=50, dispmean, dispsd){
  
  # get list of individuals and their generations
  gener<-1:ngenerations
  
  genern <- rep(1:ngenerations, times = nids)
  ID <- 1:sum(nids)
  
  # runs on generation-by-generation basis
  for(i in 1:ngenerations){
    
    id<-ID[which(genern==i)]
    dam<-rep(NA, nids[i])
    sire<-rep(NA, nids[i])
    
    Xloc<-rep(NA, nids[i])
    Yloc<-rep(NA, nids[i])
    
    # randomly allocates sex (0 = male, 1 = female)
    sex<-sample(c(0,1), length(id), replace=TRUE)
    
    # for first generation, no dams or sires are known 
    # so remain NA
    
    if(i==1){
    
      # for first generation
      # spatial locations sampled at random for X and Y coordinates
      Xloc<-sample(1:gridsize, length(id), replace=TRUE)
      Yloc<-sample(1:gridsize, length(id), replace=TRUE)
      
      # combine into single data frame
      pedigree<-data.frame(id=id, dam=dam, sire=sire, 
                           generation=i, sex=sex,
                           Xloc=Xloc, Yloc=Yloc, disp_dist=NA,
                           fall=0)
      
    }else if(i>1){
      
      # for all generations after first
      # list of all possible dams and sires
      # from previous generation
      pdams<-pedigree$id[which(pedigree$generation==(i-1) &
                                 pedigree$sex==1)]
      psires<-pedigree$id[which(pedigree$generation==(i-1) &
                                  pedigree$sex==0)]
      
      # determine number of pairs
      # depending on how many males and females
      # and the proportion of the population that is non-breeding
      npairs<-min(length(pdams), length(psires)) - 
        round(min(length(pdams), length(psires))*nonb)
      
      # selects breeding males and females
      pdams<-pedigree$id[which(pedigree$generation==(i-1) & 
                                 pedigree$sex==1 & pedigree$fall==0)]
      psires<-pedigree$id[which(pedigree$generation==(i-1) & 
                                  pedigree$sex==0 & pedigree$fall==0)]
      
      if(length(pdams)<npairs | length(psires)<npairs){
        npairs<-min(length(pdams), length(psires))
      }
      
      # selects pairs from possible dams and sires
      pairs<-data.frame(dam=sample(pdams, npairs, replace=FALSE),
                        sire=sample(psires, npairs, replace=FALSE))
      # gives each offspring their parental pair
      pairid<-as.numeric(sample(rownames(pairs), 
                                length(id), replace=TRUE))
      
      # gives each offspring their sex
      sex<-sample(c(0,1), length(id), replace=TRUE)
      
      # put into dataframe format
      addped<-data.frame(id=id, 
                         dam=pairs$dam[pairid], 
                         sire=pairs$sire[pairid],
                         generation=i, 
                         sex=sex)
      
      
      # deals with extra-pair mating (if included)
      if(!is.null(epm)){
        
        # for each individual, sample if they are extra pair
        # if 0 not extra pair
        # if 1 sire resampled from breeding population
        # if 2 dam resampled
        ext<-sample(c(0,1,2), nrow(addped), 
                    replace=TRUE, 
                    prob = c(1-epm, epm/2, epm/2))
        for(j in 1:nrow(addped)){
          if(ext[j]>0){
            if(ext[j]==1){
              addped$sire[j]<-sample(psires,1,replace=TRUE)
            }else if (ext[j]==2){
              addped$dam[j]<-sample(pdams,1,replace=TRUE)
            }
            
          }
        }
      }
      
      # Dispersal away from mother
      # First gets mother's location
      addped$Yloc<-addped$Xloc<-rep(NA, nrow(addped))
      addped$Xloc<-pedigree$Xloc[match(addped$dam, pedigree$id)]
      addped$Yloc<-pedigree$Yloc[match(addped$dam, pedigree$id)]
      addped$disp_dist<-NA
      addped$fall<-0
      
      for(j in 1:nrow(addped)){
        
          X_loc<-addped$Xloc[j]
          Y_loc<-addped$Yloc[j]
          
          # simulate the distance moved
          dist.moved<-rlnorm(1, meanlog=dispmean, sdlog=dispsd)
          # simulate angle moved
          angle<-sample(1:360, 1, replace=TRUE)
          angle<-angle * (pi/180)
          
          # get distance moved in X and Y directions
          movey<-abs(dist.moved) * sin(angle)
          movex<-abs(dist.moved) * cos(angle)
          
          # get new location of offspring
          addped$Xloc[j]<-round((X_loc + movex), digits=0)
          addped$Yloc[j]<-round((Y_loc + movey), digits=0)
          
          # dispersal distnace
          addped$disp_dist[j]<- sqrt((addped$Xloc[j] - X_loc)^2 +
                                       (addped$Yloc[j] - Y_loc)^2)
          
          # find individuals that fall off edge
          if(any(addped$Yloc[j]<1, addped$Yloc[j]>gridsize, 
                 addped$Xloc[j]<1, addped$Xloc[j]>gridsize)){
            
            addped$Yloc[j]<-addped$Xloc[j]<-NA
            addped$fall[j]<-1
            
          }
                  }
      
      # Deals with simulating missing data
      # if this is specified
      if(!is.null(missing)){ 
        # determine if mother or father is missing
        mis<-sample(c(0,1,2), nrow(addped), 
                    replace=TRUE, 
                    prob=c(1-missing, missing/2, missing/2))
        
        for(j in 1:nrow(addped)){
          if(mis[j]>0){
            if(mis[j]==1){
              addped$dam[j]<-NA
            }else if(mis[j]==2){
              addped$sire[j]<-NA
            }
          }
        }
        
      }
      
      # add new generation to the whole pedigree
      pedigree<-rbind(pedigree, addped)
    }
    
  }
  # get rid of individuals that fall off the edge
  # these are considered dead or dispersed out of population
  pedigree<-pedigree[-which(pedigree$fall==1),]
  pedigree<-pedigree[,!colnames(pedigree)%in%c("fall")]
  return(pedigree)
}


##################################
######## INPUT PARAMETERS ########
##################################
# generations to simulate
ngenerations<-10

# maximum and minimum population size
popmin<-150
popmax<-250

# spatial grid size
gridsize<-50

# dispersal parameters (for lognormal dispersal function)
dispmean<-1
dispsd<-1


##################################
######## Create PEDIGREE #########
##################################
# generate identities

ids<-sample(popmin:popmax, ngenerations, replace=TRUE)

# generate a pedigree 
ped<-pedfun(nids=ids, ngenerations=ngenerations, 
            epm=0.1, missing=NULL, nonb=0.5,
            gridsize=gridsize, dispmean, dispsd)


# make id's non-numeric
ped$id<-paste("ID",ped$id, sep="")
ped$dam[which(!is.na(ped$dam))]<-paste("ID",ped$dam[which(!is.na(ped$dam))], sep="")
ped$sire[which(!is.na(ped$sire))]<-paste("ID",ped$sire[which(!is.na(ped$sire))], sep="")

ped$id<-as.character(ped$id)
ped$dam<-as.character(ped$dam)
ped$sire<-as.character(ped$sire)
```

Number of individuals of each sex in each generation

The pedigree of the whole merpeople population, across 10 generations

# Environment

Five environment variables were simulated across the spatial(50x50) grid. Each variable was simulated with spatial autocorrelation. This was carried out using a multivariate normal random variable simulation from the R package mvnfast (Fasiolo 2016). The environmental variables therefore follow the distribution \(k \sim MVN(0, \mathbf{e^{-\phi D}})\), where \(\mathbf{e^{-\phi D}}\) is a variance-covariance matrix determined by the autocorrelation parameter \(\phi = 0.15\) and the matrix of distances between grid locations (\(\mathbf{D}\)). We use \(k\) throughout to indicate environmental variables, and \(l\) to index the 5 different environmental variables (\(1 \leq l \leq 5\)). Thus, locations that are close in space have a higher probability of similar environmental values than more distant locations (Figure ). The five environmental variables are simulated independently of one another, so show low covariance (Table 1).

```
##################################
####### Create ENVIRONMENT #######
##################################
N<-gridsize

# spatial autocorrelation of environments
phi<-0.15

# Set up all possible points on the square
simgrid <- expand.grid(1:N, 1:N)
n <- nrow(simgrid)

# Set up distance matrix
distance <- as.matrix(dist(simgrid))

# generate environmental variables
# spatial autocorrelation determined by phi and distance

X1 <- rmvn(1, rep(0, n), exp(-phi * distance))
X2 <- rmvn(1, rep(0, n), exp(-phi * distance))
X3 <- rmvn(1, rep(0, n), exp(-phi * distance))
X4 <- rmvn(1, rep(0, n), exp(-phi * distance))
X5 <- rmvn(1, rep(0, n), exp(-phi * distance))

# put into matrices 
X1 <- matrix(X1, ncol=N, nrow=N)
X2 <- matrix(X2, ncol=N, nrow=N)
X3 <- matrix(X3, ncol=N, nrow=N)
X4 <- matrix(X4, ncol=N, nrow=N)
X5 <- matrix(X5, ncol=N, nrow=N)


#### Assign environmental values based on adjusted location
ped$e_var_5<-ped$e_var_4<-ped$e_var_3<-ped$e_var_2<-ped$e_var_1<-NA

# adds a small amount of noise 
# this adds "measurement" noise
# and also means that individuals are not identical in measures
for(i in 1:nrow(ped)){
  ped$e_var_1[i]<-X1[ped$Xloc[i], ped$Yloc[i]] + rnorm(1,0,0.001)
  ped$e_var_2[i]<-X2[ped$Xloc[i], ped$Yloc[i]] + rnorm(1,0,0.001)
  ped$e_var_3[i]<-X3[ped$Xloc[i], ped$Yloc[i]] + rnorm(1,0,0.001)
  ped$e_var_4[i]<-X4[ped$Xloc[i], ped$Yloc[i]] + rnorm(1,0,0.001)
  ped$e_var_5[i]<-X5[ped$Xloc[i], ped$Yloc[i]] + rnorm(1,0,0.001)
}
```

Variance-covariance matrix of the five environmental variables

|  | Env 1 | Env 2 | Env 3 | Env 4 | Env 5 |
| --- | --- | --- | --- | --- | --- |
| Env 1 | 0.7771 | -0.0643 | -0.0008 | 0.0354 | 0.0024 |
| Env 2 | -0.0643 | 0.9049 | 0.0405 | -0.1250 | -0.0005 |
| Env 3 | -0.0008 | 0.0405 | 0.9531 | -0.1669 | 0.0314 |
| Env 4 | 0.0354 | -0.1250 | -0.1669 | 0.8979 | -0.1028 |
| Env 5 | 0.0024 | -0.0005 | 0.0314 | -0.1028 | 1.0649 |

The distribution of environmental variables across the spatial grid. Environmental values are indicated by the colours, and range between -3 and 3, due to the simulation used. Each of the five environmental variables is shown in a separate plot, with the spatial locations of the first generation of merpeople included as black points on the grid.

As mating is random and not spatially biased, mating partners are not necessarily proximate in space. This situation is similar to organisms that never move and are pollinated by vectors (e.g. plants) or that show spawning (e.g. corals). It could also be the case that individuals are stationary within a territory for most of the year, and aggregrate to breed, then return to territories (e.g. deer, pandas, fish).

# Phenotypic Traits

In the main manuscript we focus on three phenotypic traits, tail-fin colour, body size, and swimming speed, composed of different sources of variance. All individual components are simulated to have a variance of 1 and mean of 0. No covariances were simulated between any of the variance components. We first show how the different components used to generate phenotypyic traits were simulated, then how they combine to form traits.

Note that, although the three traits are made up of different components, the individual components used are the same (ie. \(\mathbf{a}\) for tail-fin colour is the same as \(\mathbf{a}\) for body size and swimming speed).

## Additive genetic effects

Additive genetic effects were simulated using the *rbv* function from the MCMCglmm package (Hadfield 2010). This uses the pedigree to simulate the breeding values, such that related individuals are more similar in their trait values than unrelated individuals. The mean breeding value was simulated to be 0, with variance of 1 (i.e. \(\mathbf{a} \sim N(0,1)\)).

```
# ADDITIVE GENETIC EFFECTS
### Get genetic portion of trait
randomA<-1
breedingValues <- rbv(ped[c("id","dam","sire")], randomA)
```

## Environmental effects

The five environmental variables described above are simulated to have linear effects on trait values, according to \(\mathbf{n} \sim N(0,1)\). To achieve a total environmental variance of one, the total variance was divided into five unequal portions (minimum 0.1, maximum 0.35) and these portions were then assigned to the five environmental variables. This therefore gave \(\sum\sigma\_{n\_{l}}^2=1\), where \(\sigma\_{n\_l}^2\) is the trait variance determined by environmental variable \(l\). Throughout, we use \(n\) to refer to trait values determined by the environment, and \(k\) to refer to the physical environmental variables themselves. Assuming that the environmental variables are uncorrelated, the total variance follows the equation:

\[
\sum\_{l=1}^5\sigma\_{n\_l}^2 = 1 = \sum\_{l=1}^{5} \beta\_{l}^{2} \sigma\_{k\_{l}}^2
\]

Where \(l\) is indexing of the environment measures (from 1 to 5), \(\beta\_{l}^2\) is the effect of the environmental variable on the trait, and \(\sigma\_{k\_l}^2\) is the variance in the environmental measure \(l\) across the whole population. To determine the value of \(\beta\_l\) for each environmental variable, we used the trait variance that had been determined to be caused by each environment:

\[
\begin{aligned}
\sigma\_{n\_l}^2 & = \beta\_{l}^{2} \sigma\_{k\_l}^{2} \\
\beta\_l^2 & = \sigma\_{n\_l}^2/\sigma\_{k\_l}^2 \\
\beta\_l & = \sqrt{ (\sigma\_{n\_l}^2/\sigma\_{k\_l}^2)}
\end{aligned}
\]

Whether the positive or negative square-root of \(\sigma\_{n\_l}^2/\sigma\_{k\_l}^2\) was taken was generated at random for each environmental variable.

The environmentally determined trait value that each individual experienced (\(n\_{il}\), where \(i\) indexes individuals and \(l\) environments) was calculated as the environmental variable experienced by the individual (\(k\_{il}\)) multiplied by the effect of that environmental variable (\(\beta\_l\)). The total environmental effect on the trait was taken as the sum of these across all five environments:

\[
\begin{aligned}
n\_{il} &= k\_{il}\beta\_{l} \\
n\_{i} &= \sum\_{l=1}^5 k\_{il}\beta\_{l}
\end{aligned}
\]

Written as a vector of all individuals:

\[
\mathbf{n} = \sum\_{l=1}^5 \mathbf{k\_l}\beta\_{l}
\]

```
# get values for betas (effects of environment on traits)
# want total variance to be 1, divided up unequally
divvyup<-function(total, portions, limit=0.35, min=0){
  
  divvys<-rep(NA, portions)
  
  for(i in 1:portions){
    if(i==1){
      divvys[i]<-runif(1, min=min, max=total*limit)
      newtotal<-total-divvys[i]
    }else if(i<portions){
    divvys[i]<-runif(1, min=min, max=newtotal*limit)
    newtotal<-newtotal-divvys[i]
    }else if(i==portions){
      divvys[i]<-newtotal
    }
    
  }
  print(sum(divvys))
    return(divvys)
}


e.var.comp<-divvyup(1, nmeasures, limit = 0.35, min=0.1)

# gets beta values to match these variances
betassq<-e.var.comp/apply(ped[grep("e_var_", colnames(ped))], 2, var)
betas<-sqrt(betassq) * sample(c(1, -1), 5, replace=T)

# gets trait values based on individual's environment
trait_env<-t(t(ped[grep("e_var_", colnames(ped))])*betas)
e_tr<-rowSums(trait_env)
```

## Maternal effects

**Maternal genetic effects** were generated in a manner akin to the additive genetic effects (breeding values) described above. Individual breeding values for a maternal effect (the effect an individual would have on its own offspring) were simulated using the *rbv* function, with a mean=0 and variance=1. A mother’s maternal effect was then assigned to her offspring, thereby giving the effect of mothers on offspring trait values (maternal genetic effects \(\mathbf{Ma}\)). For individuals without mothers in the pedigree, values were randomly sampled from a normal distribution with variance 1 and mean 0.

```
### MATERNAL GENETIC
mat_bv <- rbv(ped[c("id","dam","sire")], 1)
# assign values that mothers express

P_mat_bv<-mat_bv[match(ped$dam, rownames(mat_bv))]

# get random values for individuals missing mothers
P_mat_bv[which(is.na(P_mat_bv))]<-rnorm(length(P_mat_bv[which(is.na(P_mat_bv))]), 0, 1)
```

**Maternal environmental effects** were generated in the same way as direct environmental effects, with a variance of 1 and mean 0. As before, variance was divided up unequally between the five environemtnal variables, and linear effects (\(\beta\_l\)) were calculated. The maternal effect an mother expressed was then calculated as the mother’s environment multipled by the linear effect of that environment (\(\beta\_{l}k\_{l,j}\)), summed across all five environments. As with the maternal genetic effect, these values were then assigned to the offspring of the mothers, generating maternal environmental effects for all individuals (\(\mathbf{Mn}\)). For individuals without mothers in the pedigree, values were randomly sampled from a normal distribution with variance \(\sigma\_{Mn}^{2}\) and mean 0.

```
### MATERNAL ENVIRONMENT VALS 

# as with direct effect, total variance is 1
# partitioned unequally between environmental effects
e.var.comp_m<-divvyup(1, nmeasures, limit = 0.35, min=0.1)

# get effects on traits
betassq_m<-e.var.comp_m/apply(ped[grep("e_var_", colnames(ped))], 2, var)
betas_m<-sqrt(betassq_m) * sample(c(1, -1), 5, replace=T)

# get mothers own effect on offspring
trait_m_env<-t(t(ped[grep("e_var_", colnames(ped))])*betas_m)

mat_e_tr<-rowSums(trait_m_env)
names(mat_e_tr)<-ped$id

# get maternal effect experienced
P_mat_e<-mat_e_tr[match(ped$dam, names(mat_e_tr))]
P_mat_e[which(is.na(P_mat_e))]<-rnorm(length(P_mat_e[which(is.na(P_mat_e))]), 0, sqrt(var(P_mat_e, na.rm=TRUE)))
```

## Residual variance

Residual variance (\(\mathbf{r}\)) was simulated as a random normal distribution with mean 0 and variance 1 (\(r \sim N(0,1)\)). Residual variance was uncorrelated between individuals.

```
### Residual noise
resid<-rnorm(nrow(ped), 0, 1)
```

## Phenotypic trait values

For each trait, the individual trait values are calculated as the sum of the components that compose the trait:

**Tail-fin colour** is composed of an additive genetic effect (\(\mathbf{a}\)), the direct effects of the five environmental variables (\(\mathbf{n}\)), and a random residual effect (\(\mathbf{r}\)).

\[
\mathbf{z\_{1}} = \mathbf{a} + \mathbf{n} + \mathbf{r}
\]

```
# Tail-fin colour - additive genetic and direct environmental effects
ped$trait_ae <- breedingValues + e_tr + resid
```

**Body size** is composed of an additive genetic effect (\(\mathbf{a}\)), the direct effects of the five environmental variables (\(\mathbf{n}\)), a maternal environmental effect (\(\mathbf{Mn}\)), and a random residual effect (\(\mathbf{r}\)).

\[
\mathbf{z\_{2}} = \mathbf{a} + \mathbf{n} + \mathbf{Mn} + \mathbf{r}
\]

```
# Body size - additive genetic, direct and maternal environmental effects
ped$trait_mee <- breedingValues + e_tr + P_mat_e + resid
```

**Swimming speed** is composed of an additive genetic effect (\(\mathbf{a}\)), the direct effects of the five environmental variables (\(\mathbf{n}\)), a maternal environmental effect (\(\mathbf{Mn}\)), a maternal genetic effect (\(\mathbf{Ma}\)), and a random residual effect (\(\mathbf{r}\)).

\[
\mathbf{z\_{3}} = \mathbf{a} + \mathbf{Ma} + \mathbf{n} + \mathbf{Mn} + \mathbf{r}
\]

```
# Swimming speed - additive and maternal genetic, direct and maternal environmental effects
ped$trait_mgee<- breedingValues + e_tr + P_mat_bv + P_mat_e + resid
```

# Epi-alleles

Epigenetic marks across the genome were simulated as methylation islands, such that at each locus there was a variable number of CpG sites that were methylated or non-methylated.

Three such types of methylation island were simulated:

**Environmentally derived, non-heritable**. We simulated 100 epigenetic islands that varied depending on the environment an individual itself experienced (20 per environmental variable). In each case, the maximum number of methylations in an island was 20 (minimum 0, as there cannot be negative numbers of methylations). The linear effect of the environment (\(k\_{l}\)) on each epigenetic mark was generated as:

\[
b\_{l,p} = \pm \frac{20}{max(\mathbf{k\_{l}})-min(\mathbf{k\_{l}})} - N(0,1)
\]

Where \(p\) indexes the epigenetic islands and \(j\) the environmental variables. The direction of the effect (positive or negative) was chosen at random for each of the 1 to \(p\) epigenetic islands.

Individual epigenetic marks were then generated as \(b\_{l,p}\*e\_{il} + N(0,1)\). \(N(0,1)\) is used in each case to add random noise to measures.

**Environmentally derived, partially heritable**. Thirty epigenetic islands (6 per environmental variable) were simulated that had partially heritable environmental effects. The number of CpG sites that were methylated within an island could be maternally inherited, based on the environment the mother herself experienced. The effects of the environmental variables on epigenetic marks (\(b\_{l,p}\)) was calculated as above. The methylation levels determined by the individual’s environment and by the environment were calculated. The actual methylation experienced by the individual was then selected from these two options, according to the rate of epigenetic reset (0.5 in all cases), so that there was a 50% chance that an individual inherited methylation marks from its mother.

Both the heritable and non-heritable environmental epialleles were generated using the following function:

```
### Function to create methylation islands, based on environment 

# id  = id vector
# dam = dam vector
# environment =  environmental variable
# effect = the direction of effect of the environment 
#           (positive, negative, or random)
# dir.effect = optional vector specifying the direction of effects 
#               on each epigenetic mark 
#               (values should be 1 or -1 to specify positive and 
#               negative, respectively)
# reset =  the rate of reset between generations. 
#          1= total reset , 0 =no reset
# noise.sd = the amount of noise around the relationship 
#             between environmental value and marks
# nepi = number of epigenetic islands being created
# islandmax = the maximum number of methylation marks at a given island

# Assumes absolute reset :
#   methylation is either the pattern inherited based on the mother's
#   environment, or is reset based on the environment the individual is in.

methylation<-function(id, mother, environment, 
                      effect="positive",
                      dir.effect=NULL,
                      reset=1,
                      noise.sd=1,
                      nepi=1, 
                      islandmax=20){

# set up data frame    
epimarks <- as.data.frame(matrix(NA, ncol=nepi, nrow=length(id)))  
colnames(epimarks) <- c(paste("epi",1:nepi,sep="."))  
rownames(epimarks) <- id

if(length(islandmax)==1){
  islandmax<-rep(islandmax, nepi)
}

# gets the strength and direction of effect of the environment
# for each mark
midpoint <- islandmax/2

envmax <- rep(max(abs(environment)), nepi)

d<-diff(range(environment))

b <- (islandmax)/d  + rnorm(nepi, mean=0, sd=1)

if(is.null(dir.effect)){
if(effect=="random") dir.effect<-sample(c(1,-1),nepi, replace=TRUE)

if(effect=="negative") dir.effect<-rep(-1, nepi)
if(effect=="positive") dir.effect<-rep(1, nepi)

}else if(!is.null(dir.effect) & length(dir.effect)==1 & nepi>1){
  dir.effect<-rep(dir.effect, nepi)
}

b<-b*dir.effect


# get marks
for(i in 1:length(id)){
  for(j in 1:nepi){
# for individuals with no dam, have to sample environment  
  if(is.na(mother[i])){
    epimarks[i,j]<- round(midpoint[j] + environment[i] * b[j] + round(rnorm(1, mean=0, sd=noise.sd)))
  }else if(!is.na(mother[i])){
    
    dam<-mother[i]
    damenv<-environment[which(id==as.character(dam))]
    
    epidam<- round(midpoint[j] + damenv * b[j] + round(rnorm(1, mean=0, sd=noise.sd)))
    epiown<- round(midpoint[j] + environment[i] * b[j] + round(rnorm(1, mean=0, sd=noise.sd)))
    
   
    epimarks[i,j]<-sample(c(epidam, epiown), size=1, prob=c(1-reset, reset))

       
  }
  if(epimarks[i,j]<0){print(cat( rownames(epimarks[i,]), "Value less than 0 found, replaced with 0"))
    
    epimarks[i,j]<-0
  }
    if(epimarks[i,j]>20){print(cat( rownames(epimarks[i,]), "Value more than 20 found, replaced with 20"))
      
      epimarks[i,j]<-20
    }
}  
}  

return(epimarks)
}
```

Actual values are then generated by varying the reset value. For non-heritable, reset=1 (i.e. always reset to values predicted by an individuals *own* environment). For heritable, reset=0.5.

```
# directly affected by relevant environmental variables  
  e_1<-methylation(ped$id, ped$dam, ped$e_var_1, 
                   effect="random",
                   reset=1,
                   noise.sd=1,
                   nepi=nloci_direct/5, 
                   islandmax=20)    
  colnames(e_1)<-paste("e1", colnames(e_1), sep=".")
  
  e_2<-methylation(ped$id, ped$dam, ped$e_var_2, 
                   effect="random",
                   reset=1,
                   noise.sd=1,
                   nepi=nloci_direct/5, 
                   islandmax=20)
  colnames(e_2)<-paste("e2", colnames(e_2), sep=".")
  
  e_3<-methylation(ped$id, ped$dam, ped$e_var_3, 
                   effect="random",
                   reset=1,
                   noise.sd=1,
                   nepi=nloci_direct/5, 
                   islandmax=20)
  colnames(e_3)<-paste("e3", colnames(e_3), sep=".")
  
  e_4<-methylation(ped$id, ped$dam, ped$e_var_4, 
                   effect="random",
                   reset=1,
                   noise.sd=1,
                   nepi=nloci_direct/5, 
                   islandmax=20)
  colnames(e_4)<-paste("e4", colnames(e_4), sep=".")
  
  e_5<-methylation(ped$id, ped$dam, ped$e_var_5, 
                   effect="random",
                   reset=1,
                   noise.sd=1,
                   nepi=nloci_direct/5, 
                   islandmax=20)  
  colnames(e_5)<-paste("e5", colnames(e_5), sep=".")

  
# affected by maternal environmental variables (with reset to own environment)    
e_m1<-methylation(ped$id, ped$dam, ped$e_var_1, 
                  effect="random",
                  reset=m_reset,
                  noise.sd=1,
                  nepi=nloci_inherited/5, 
                  islandmax=20)    
colnames(e_m1)<-paste("em1", colnames(e_m1), sep=".")

e_m2<-methylation(ped$id, ped$dam, ped$e_var_2, 
                  effect="random",
                  reset=m_reset,
                  noise.sd=1,
                  nepi=nloci_inherited/5, 
                  islandmax=20)
colnames(e_m2)<-paste("em2", colnames(e_m2), sep=".")

e_m3<-methylation(ped$id, ped$dam, ped$e_var_3, 
                  effect="random",
                  reset=m_reset,
                  noise.sd=1,
                  nepi=nloci_inherited/5, 
                  islandmax=20)
colnames(e_m3)<-paste("em3", colnames(e_m3), sep=".")

e_m4<-methylation(ped$id, ped$dam, ped$e_var_4, 
                  effect="random",
                  reset=m_reset,
                  noise.sd=1,
                  nepi=nloci_inherited/5, 
                  islandmax=20)
colnames(e_m4)<-paste("em4", colnames(e_m4), sep=".")

e_m5<-methylation(ped$id, ped$dam, ped$e_var_5, 
                  effect="random",
                  reset=m_reset,
                  noise.sd=1,
                  nepi=nloci_inherited/5, 
                  islandmax=20)
colnames(e_m5)<-paste("em5", colnames(e_m5), sep=".")
```

**Non-environmental, non-heritable.** One hundred methylation islands were created that were not affected by the simulated environmental variables, nor were they heritable. For each of these islands, the maximum number of methylated CpG sites for each island was simulated to be between 4 and 20 sites. The probabilities of different marks was then generated using the function, with the mean value being half the maximum number of methylated CpG sites, and the standard deviation sampled at random between the values 0 and 10. Using these probabilities, each individual then sampled methylation between 0 and and the maximum island size. This was repeated independently for each of the 100 methlyation islands.

```
# unaffected by environment and not inherited 
# (random marks)

e_rand<-as.data.frame(matrix(NA, 
                             ncol=nloci_random, 
                             nrow=length(ped$id)))
colnames(e_rand)<-paste("r.epi",1:nloci_random, sep=".")
rownames(e_rand)<-ped$id


for(i in 1:nloci_random){
  # determin size of island (between 4 and 20 total CpG points that
  # can be methylated)
  islandsize<-sample(seq(4,20,1), 1)
  # how much variation (around the midpoint) will there be in marks
  sd<-runif(1, min=0, max=10)
  
  # find probabilities of different numbers of methylations
  ps<-dnorm(0:islandsize, islandsize/2, sd)

  # sample from these probabilities
  epirand<-sample(0:islandsize, nrow(ped), replace=TRUE, prob=ps)

  e_rand[,i]<-epirand
  
  
}
```

# Social Network

A social network was created across the dataset, using information from both the spatial location of individuals as well as the pedigree. Within a generation, the probability of connection (and the strength of connection) between a given pair of individuals was dependent on the spatial distance between those individuals.

Between adjacent generations, there was a lower probability of connections, although the probability of connection remained affected by the spatial distance. Intergenerational connections were most likely between mother-offspring pairs. No connections were simulated between non-adjacent generations, as the semelparous nature of the population means that these individuals would not come into contact.

The code to generate the social network was adapted from that provided by Matthew Silk, given below:

```
#Code to create spatial and trait-driven networks


#this function makes social networks according to spatial locations

# pedigree = pedigree in order "id", "dam", "sire"
# generation = the generation an individual is found in 
# spatialx = x coordinate of individual's location
# spatialy = y coordinate of individual's location

#d.eff = effect of distance on likelihood of connection (within generation)
#i.dens =  influences likelihood of within generation connections 
#m.dens = influences likelihood of mother-offspring connections
#p.dens = influences likelihood of father-offspring connections
#g.dens =  influences likelihood of random between-generation connections
#g.eff = effect of distance on likelihood of connection between generations

#set plot=T to look at the network

network.generator<-function(pedigree,
                            generation,
                            spatialx, spatialy,
                            d.eff, i.dens,
                            m.dens, p.dens, g.dens, g.eff,
                            plot=F){

#create the individuals
indivs<-pedigree$id

#arrange in data frame and order by group
inds<-data.frame(indivs,generation,pedigree$dam,pedigree$sire)


#create dataframe of spatial information
spat<-data.frame(indivs,spatialx,spatialy)

#create distance matrix for groups
dists<-as.matrix(dist(spat[,2:3]))
rownames(dists)<-colnames(dists)<-indivs

#standardise and invert (so 1 is closest and 0.001 is furthest away)
dists2<-dists/max(dists)
dists3<-1.001-dists2
diag(dists3)<-1

#-----------------------------------------------------------------------------------------------------------------

#####NETWORK STUFF#####
#create empty network in association matrix form
net.d<-array(NA,dim=rep(nrow(inds),2))
colnames(net.d)<-rownames(net.d)<-inds[,1]

for (i in 1:(nrow(inds)-1)){
  
   for(j in (i+1):nrow(inds)){
     
     # INDIVIDUALS IN SAME GENERATION
     
      if(inds[,2][i]==inds[,2][j]){
        
         net.d[i,j]<-round(rnbinom(1,size=i.dens*(dists3[which(colnames(dists3)==inds[,1][i]),which(colnames(dists3)==inds[,1][j])])^d.eff,prob=0.3))+
           round(rnbinom(1,size=i.dens*(dists3[which(colnames(dists3)==inds[,1][i]),which(colnames(dists3)==inds[,1][j])])^d.eff,prob=0.3))
         
      }else if(abs(inds[,2][i]-inds[,2][j])==1){
        
      # INDIVIDUALS IN ADJACENT GENERATIONS  
        if(!is.na(inds[,3][j]) & 
               as.character(inds[,1][i])==as.character(inds[,3][j]) & 
               m.dens>0){
        
        
        #MATERNAL links
        net.d[i,j]<-round(rnbinom(1,size=m.dens*(dists3[which(colnames(dists3)==inds[,1][i]),which(colnames(dists3)==inds[,1][j])])^(d.eff+g.eff),prob=0.3))+round(rnbinom(1,size=m.dens*(dists3[which(colnames(dists3)==inds[,1][i]),which(colnames(dists3)==inds[,1][j])])^(d.eff+g.eff),prob=0.3))
        
      }else if(!is.na(inds[,3][j]) & 
               as.character(inds[,1][i])==as.character(inds[,4][j]) & 
               p.dens>0){
     
        #PATERNAL links
        net.d[i,j]<-round(rnbinom(1,size=p.dens*(dists3[which(colnames(dists3)==inds[,1][i]),which(colnames(dists3)==inds[,1][j])])^(d.eff+g.eff),prob=0.3))+round(rnbinom(1,size=p.dens*(dists3[which(colnames(dists3)==inds[,1][i]),which(colnames(dists3)==inds[,1][j])])^(d.eff+g.eff),prob=0.3))
        
      }else{
        #RANDOM links
        net.d[i,j]<-round(rnbinom(1,size=g.dens*(dists3[which(colnames(dists3)==inds[,1][i]),which(colnames(dists3)==inds[,1][j])])^(d.eff+g.eff),prob=0.3))+round(rnbinom(1,size=g.dens*(dists3[which(colnames(dists3)==inds[,1][i]),which(colnames(dists3)==inds[,1][j])])^(d.eff+g.eff),prob=0.3))
      }
        
        # NO OTHER LINKS
      } else if(abs(inds[,2][i]-inds[,2][j])!=1){
        net.d[i,j]<-0
      }
      }
   }


#this sets the diagonal to zero i.e. no self loops in network
net3.d<-t(net.d)
diag(net3.d)<-0

#this symmetrises the association matrix
for (i in 1:nrow(inds)){
for (j in 1:nrow(inds)){
if(j<i){
net3.d[j,i]<-net.d[j,i]
}
}
}


#plots the graph of each network made if plot=TRUE
if(plot==T){
   dev.new()
   par(mfrow=c(1,1))
   net2.d<-graph.adjacency(net.d,mode="undirected",weighted=TRUE,diag=FALSE)
   net2.d<-set.vertex.attribute(net2.d, "group", index=V(net2.d), inds[,2])
   V(net2.d)$group
   #V(net2.d)$color=V(net2.d)$group #assign the "Group" attribute as the vertex color
   V(net2.d)$color="blue"
   plot(net2.d,edge.width=(E(net2.d)$weight)^0.25,layout=layout.fruchterman.reingold(net2.d),vertex.size=8,vertex.label=NA,margin=c(0,0,0,0))
}

#fill in lists with network data
net4.d<-net3.d
for(i in 1:nrow(net4.d)){
for(j in 1:ncol(net4.d)){
if(is.na(net4.d[i,j])==TRUE){
net4.d[i,j]<-0
}
}
}

pop.dat<-list(inds,net4.d)

return(pop.dat)

#end function
}
```

```
# set parameters
d.eff=11
g.eff=5
i.dens=0.3
p.dens=0
m.dens=0.6
g.dens=0.1

# simulate network
simNet<-network.generator(ped[,c("id","dam","sire")], ped$generation, 
                          spatialx=ped$Xloc, spatialy=ped$Yloc,
                          d.eff=d.eff, g.eff=g.eff, 
                          o.dens=o.dens, i.dens=i.dens, 
                          m.dens=m.dens, p.dens=p.dens, 
                          g.dens=g.dens,
                          plot=F)

# get the data wanted for later analysis
n2<-simNet[[2]]
```

Fasiolo, M. (2016). An introduction to mvnfast. R package version 0.1.6.

Hadfield, J.D. (2010). MCMC methods for multi-response generalized linear mixed models: the MCMCglmm R package. *Journal of Statistical Software*, **33**, 1–22.
