# Supplementary material for: A guide to using a multiple-matrix animal model to disentangle genetic and nongenetic causes of phenotypic variance
Source: PLoS One. 2018 Oct 12;13(10):e0197720. doi: 10.1371/journal.pone.0197720 (PMC6193571; doi:10.1371/journal.pone.0197720)
Supplement: S3 File — Supplementary analyses using reduced data sizes to show the effect on estimation of variance components. (DOCX) [file pone.0197720.s003.docx]

***A guide to using a multiple-matrix animal model to disentangle genetic and non-genetic causes of phenotypic variance***

**Supplementary material: Data requirements**

For the animal models that contain both genetic and nongenetic similarity matrices to partition variance components, there needs to be both sufficient data and data with sufficient contrasts to separate these effects. The exact data requirements to run these models will be dependent upon multiple factors, including the true underlying sources of variance, pedigree structure, and the model that is to be specified (e.g. whether parental environmental effects are to be included or not). In those papers that have estimated variance attributable to spatial overlap sample sizes for most traits were large, and a single spatial overlap matrix was included in the models. For red deer, there were over 700 individuals with spatial overlap for the trait with the least data^1^. Data from over 1000 individuals were used to assess spatial overlap in song sparrows^2^. Finally, for Soay sheep, over 1000 individuals were included for models involving lamb traits (which use the maternal spatial overlap to estimate spatial effects), but 300 or fewer samples for adult morphological traits (which use an individual's *own* spatial overlap)^3^.

Given the different sample sizes used across studies that have included spatial overlap matrices, it is challenging to get an overview of how much data is required to get accurate variance estimates. To address this, we provide a qualitative test of data requirements shown for the (full) models and traits demonstrated in the main manuscript. Thus, for each trait we show the model includes all simulated components of variance. To test the data required for these models, we reran models with reduced datasets in two ways. Firstly, we investigated the minimum number of generations required to produce accurate results. We sequentially deleted generations from the data (initial number of generations=10, with generations numbered 1 to 10), down to 3 generations. In each case, we retained earlier generations and removed the last *n* generations in time (so if two generations were removed, we kept generations 1 to 8).

Secondly, we investigated the tolerable level of missing phenotypic and/or pedigree data. For this, we deleted random subsets of the data (from 5% to 75% deleted, each proportion tested 5 times). Where an individual no longer had a parent present in the dataset due to their removal, the sire or dam for that individual was considered missing. Thus, this deletion of data also affected the completeness of the pedigree. We qualitatively evaluated the performance of the models based on changes in variance estimates and standard errors. It should be noted that the analysis here is specific to the dataset simulated, and different results might be expected with different populations. The rate of extra-pair paternity, the ratio of full- to half-siblings, the rate of dispersal, and many other factors could affect the actual data requirements. We therefore recommend that researchers interested in these models run power analyses with their own datasets.

The original dataset (with no deletion) had 1806 individuals.

| **Number of generations removed** | **Number of individuals remaining** |
| --- | --- |
| 0 | 1806 |
| 1 | 1616 |
| 2 | 1471 |
| 3 | 1314 |
| 4 | 1109 |
| 5 | 882 |
| 6 | 706 |
| 7 | 512 |
| **Proportion of data removed** |  |
| 0.00 | 1806 |
| 0.05 | 1716 |
| 0.15 | 1536 |
| 0.25 | 1355 |
| 0.35 | 1174 |
| 0.45 | 994 |
| 0.55 | 813 |
| 0.65 | 633 |
| 0.75 | 452 |

**Trait 1 – Tail-fin colour**

Tail-fin colour was simulated to be affected by an additive genetic effect (*a*), environmental effects (*n)*, and a residual effect *(r).* Thus, each individual’s trait value is determined by:

$$y_{i}=a_{i}+n_{i}+r_{i}$$

The full model for this trait estimates phenotypic variance that is partitioned into additive genetic, environmental, and residual variances.

Figure 1 shows the results as increasing numbers of generations of individuals are removed from the data. Estimates remain relatively stable up to the removal of 5 generations, although the estimation of *V_n_* increases somewhat, and error bars increase in size. With the removal of six and seven generations of data, the estimates of *V_n_* increases substantially. When six generations are removed (four remain), the data includes ~700 individuals.

Figure 2 shows the results from models as random subsets of data are deleted. Variance estimates remain relatively stable with less than 35% of data removed, although there is some instability in the estimation of *V_n_*. The values become much less stable after more than 45% of data is removed, with much larger error bars. Thus, the values appear to become much less well estimated when the data contains fewer than 1100 individuals.

Thus, for the data structure of this simulation, more than 700 samples are likely to be necessary to generate good estimates, with more samples needed if the data is less complete (e.g. missing parents in the pedigree).

**
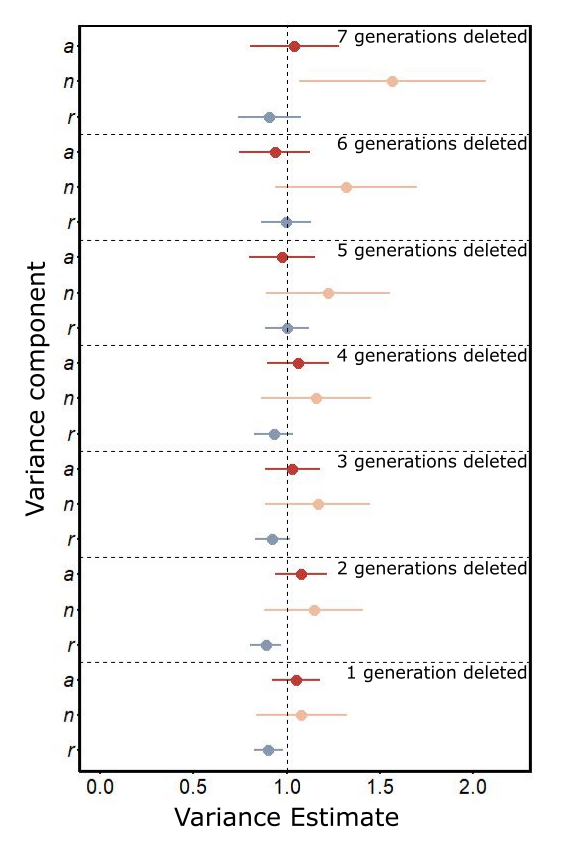
**

Figure 1: Graph showing variance estimates from animal models. In each sub-graph, different numbers of generations have been deleted from the data, as indicated. Points show the estimates of variance components, and error bars their standard errors.

**
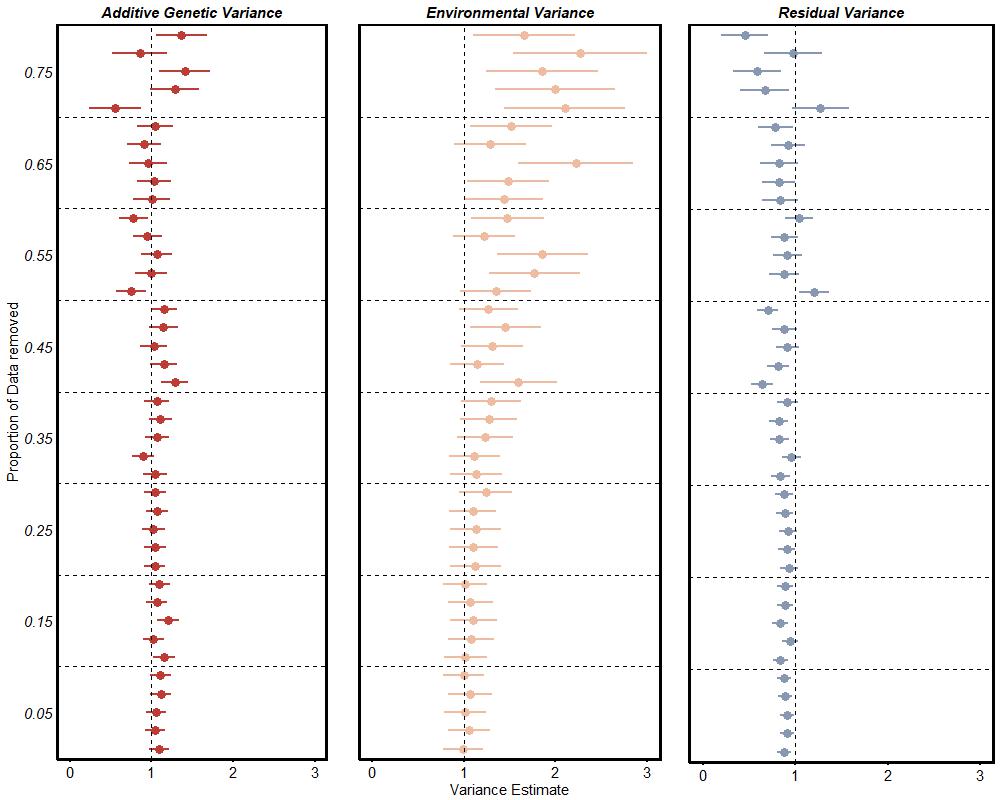
**

Figure 2: Graph showing variance estimates from animal models, when random subsets of the data have been removed. y-axes indicate the proportion of data that has been removed. Each proportional deletion is repeated 5 times

**Trait 2 – body size**

Body size was simulated to be affected by an additive genetic effect (*a*), environmental effects (*n)*, maternal environmental effects (*Mn*), and a residual effect *(r).* Thus, each individual’s trait value is determined by:

$$y_{i}=a_{i}+n_{i}+{Mn}_{ij}+r_{i}$$

The full model for this trait estimates phenotypic variance that is partitioned into additive genetic, environmental, maternal environmental, and residual variances.

Figure 3 shows the results from models as increasing numbers of generations of individuals are removed from the data. The estimation of *V_n_* is relatively consistent up to the removal of 4 generations, although the standard errors increase, as is also the case for the additive genetic and residual variances. The maternal environmental variance becomes less stable with the removal of 3 or more generations (fewer than 1300 individual).

Figure 4 shows the results from models as random subsets of data are deleted. Estimates of both *V_n_* and *V_Mn_* become larger and more unstable with the removal of 15% or more of the data (fewer than 1700 individuals), and increased instability is seen in the other variance components.

As for the effects of data removal on tail-fin colour variance estimates, the differences between the two types of data removal are likely to be driven by the loss of information about mothers when random data is deleted, rather than by generation. Nevertheless, this shows that when maternal environmental variance exists and is to be estimated, the data requirements are higher than if only direct variances are estimated.

**
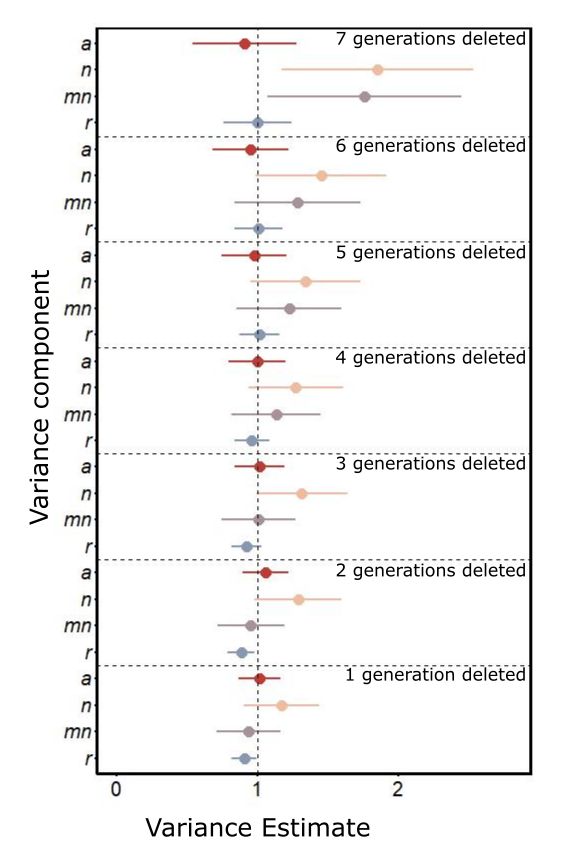
**

Figure 3: Graph showing variance estimates from animal models. In each sub-graph, different numbers of generations have been deleted from the data, as indicated. Points show the estimates of variance components, and error bars their standard errors.


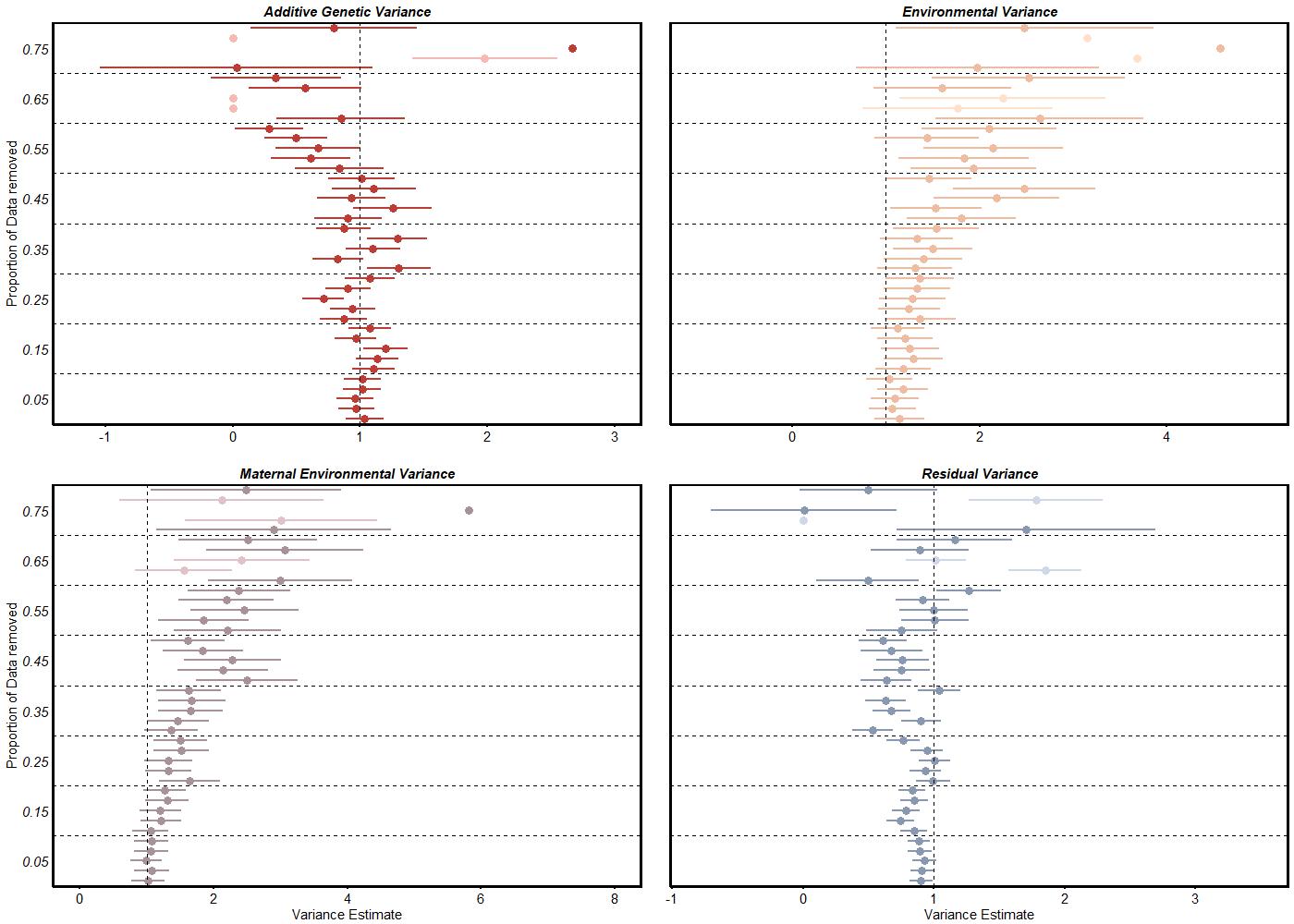


Figure 4: Graph showing variance estimates when random subsets of the data have been removed, with each proportional deletion repeated 5 times. Paler points indicate models where likelihood has not converged, or some estimates have gone to the boundary

**Trait 3 – swimming speed**

Swimming speed was simulated to be affected by an additive genetic effect (*a*), a maternal genetic effect (M*a*), environmental effects (*n)*, maternal environmental effects (*Mn*), and a residual effect *(r).* Thus, each individual’s trait value is determined by:

$$y_{i}=a_{i}+{Ma}_{ij}+n_{i}+{Mn}_{ij}+r_{i}$$

The full model for this trait estimates phenotypic variance that is partitioned into additive genetic, maternal genetic, environmental, maternal environmental and residual variances. As shown in the main text, this model struggles to completely partition maternal genetic and environmental effects when all data is present, so *a priori* we know that more data is required to accurately partition these components. Nevertheless, data removal will give us an idea of the severity of issues when even less data are available.

Figure 5 shows the results from models as increasing numbers of generations of individuals are removed from the data. Although the maternal components are misestimated in all cases, these remain relatively stable up to (and including) the deletion of 3 generations of data, as do other variance components. With increasing numbers of generations removed, variance components (particularly maternal) are less well estimated, and standard errors increase.

Figure 6 shows the results from models as random subsets of data are deleted. Estimation of components becomes less stable with the removal of 15% or more of the data (leaving 1500 individuals or less), particularly the two maternal components.

Thus, extensive datasets are needed to estimate both maternal genetic and maternal environmental variances.


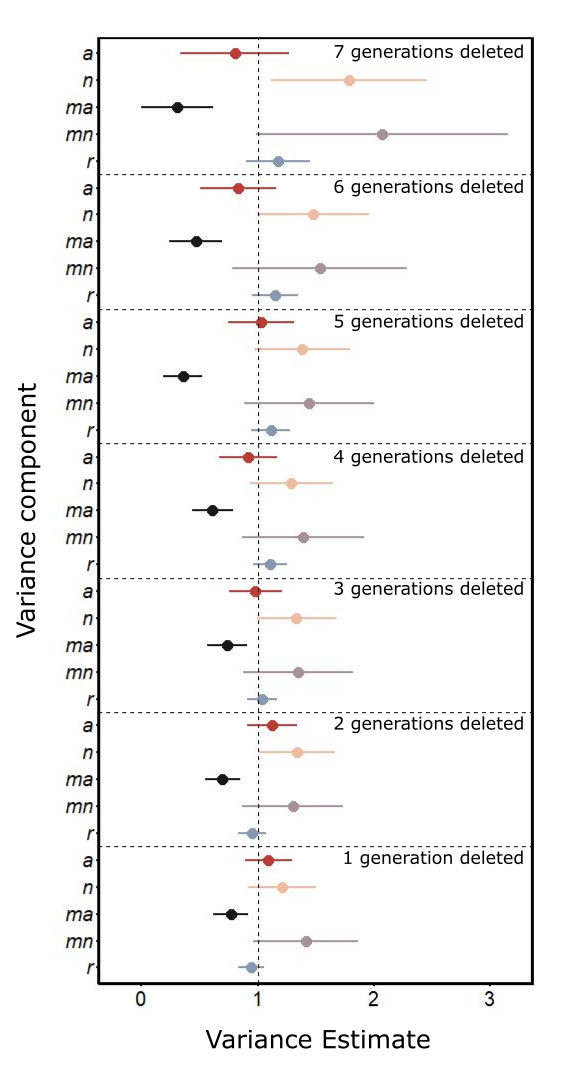


Figure 5: Graph showing variance estimates, with different numbers of generations removed from the data. Points show estimates, and bars their standard errors.


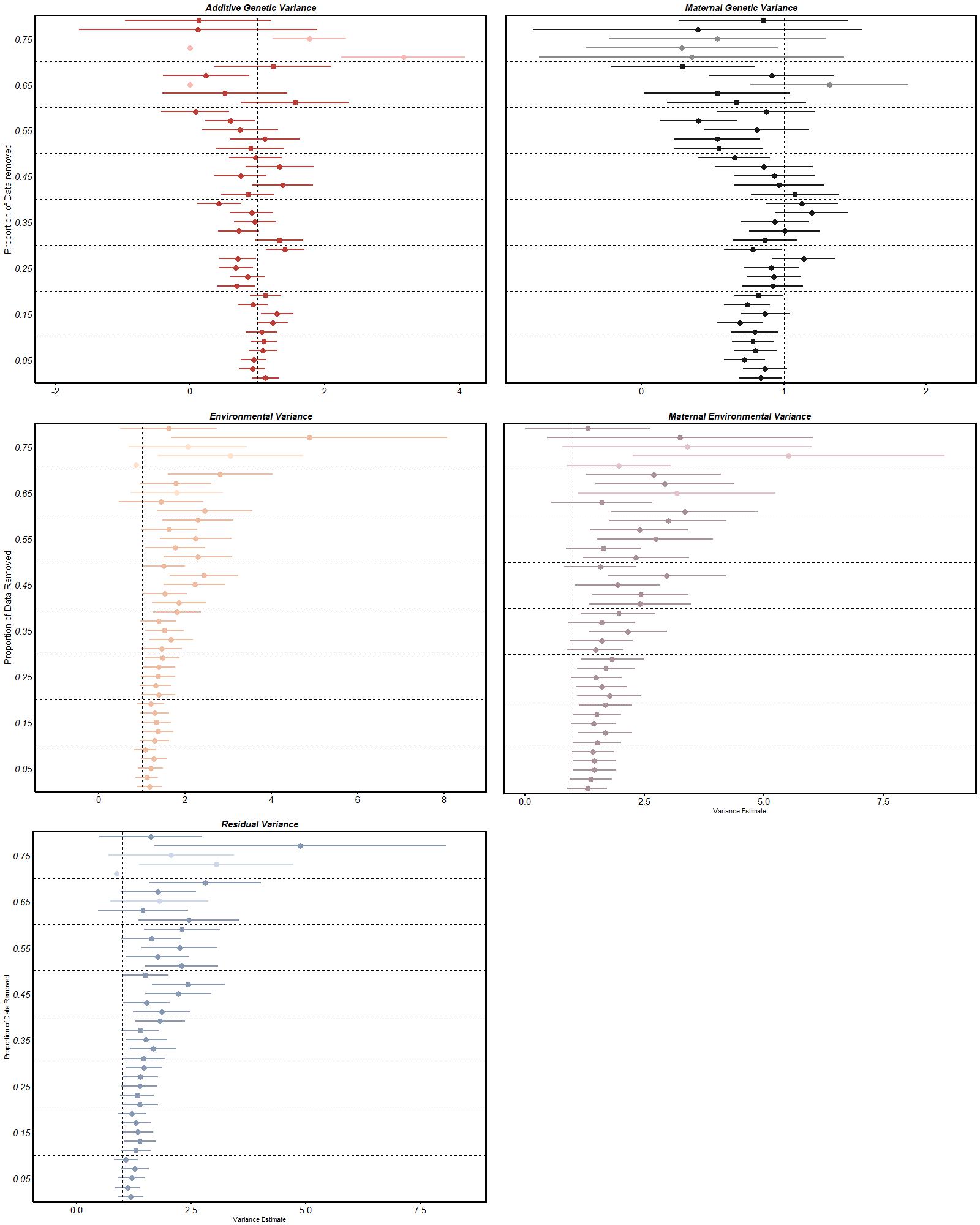


Figure 6: Graph showing variance estimates when random subsets of the data have been removed. Paler points indicate models where likelihood has not converged, or some estimates have gone to the boundary

**References**

(1) Stopher, K. V; Walling, C. A.; Morris, A.; Guinness, F. E.; Clutton-Brock, T. H.; Pemberton, J. M.; Nussey, D. H. *Evolution (N. Y).* **2012**, *66*, 2411–2426.

(2) Germain, R. R.; Wolak, M. E.; Arcese, P.; Losdat, S.; Reid, J. M. *J. Anim. Ecol.* **2016**, *85* (6), 1613–1624.

(3) Regan, C. E.; Pilkington, J. G.; Berenos, C.; Pemberton, J. M.; Smiseth, P. T.; Wilson, A. J. *J. Evol. Biol.* **2017**, *30*, 96–111.
